# Microbiota-Derived Short-Chain Fatty Acids Modulate Expression of *Campylobacter jejuni* Determinants Required for Commensalism and Virulence

**DOI:** 10.1128/mBio.00407-17

**Published:** 2017-05-09

**Authors:** Paul M. Luethy, Steven Huynh, Deborah A. Ribardo, Sebastian E. Winter, Craig T. Parker, David R. Hendrixson

**Affiliations:** a Department of Microbiology, University of Texas Southwestern Medical Center, Dallas, Texas, USA; b Produce Safety and Microbiology, USDA Agricultural Research Service, Albany, California, USA; Loyola University Chicago; University of Hawaii at Manoa

**Keywords:** *Campylobacter jejuni*, commensalism, intestinal colonization, short-chain fatty acids, transcriptional regulation

## Abstract

*Campylobacter jejuni* promotes commensalism in the intestinal tracts of avian hosts and diarrheal disease in humans, yet components of intestinal environments recognized as spatial cues specific for different intestinal regions by the bacterium to initiate interactions in either host are mostly unknown. By analyzing a *C. jejuni* acetogenesis mutant defective in converting acetyl coenzyme A (Ac-CoA) to acetate and commensal colonization of young chicks, we discovered evidence for *in vivo* microbiota-derived short-chain fatty acids (SCFAs) and organic acids as cues recognized by *C. jejuni* that modulate expression of determinants required for commensalism. We identified a set of *C. jejuni* genes encoding catabolic enzymes and transport systems for amino acids required for *in vivo* growth whose expression was modulated by SCFAs. Transcription of these genes was reduced in the acetogenesis mutant but was restored upon supplementation with physiological concentrations of the SCFAs acetate and butyrate present in the lower intestinal tracts of avian and human hosts. Conversely, the organic acid lactate, which is abundant in the upper intestinal tract where *C. jejuni* colonizes less efficiently, reduced expression of these genes. We propose that microbiota-generated SCFAs and lactate are cues for *C. jejuni* to discriminate between different intestinal regions. Spatial gradients of these metabolites likely allow *C. jejuni* to locate preferred niches in the lower intestinal tract and induce expression of factors required for intestinal growth and commensal colonization. Our findings provide insights into the types of cues *C. jejuni* monitors in the avian host for commensalism and likely in humans to promote diarrheal disease.

## IMPORTANCE

*Campylobacter jejuni* is a commensal of the intestinal tracts of avian species and other animals and a leading cause of diarrheal disease in humans. The types of cues sensed by *C. jejuni* to influence responses to promote commensalism or infection are largely lacking. By analyzing a *C. jejuni* acetogenesis mutant, we discovered a set of genes whose expression is modulated by lactate and short-chain fatty acids produced by the microbiota in the intestinal tract. These genes include those encoding catabolic enzymes and transport systems for amino acids that are required by *C. jejuni* for *in vivo* growth and intestinal colonization. We propose that gradients of these microbiota-generated metabolites are cues for spatial discrimination between areas of the intestines so that the bacterium can locate niches in the lower intestinal tract for optimal growth for commensalism in avian species and possibly infection of human hosts leading to diarrheal disease.

## INTRODUCTION

*Campylobacter jejuni* is a commensal bacterium of the intestinal tracts of avian species and many other animals in the wild and in agriculture. As a consequence of these zoonotic infections, poultry and other meats in the human food supply are frequently contaminated with *C. jejuni*. Consumption of these contaminated meats may culminate in an inflammatory diarrheal disease in humans. As such, *C. jejuni* is a leading cause of bacterial diarrheal disease in humans throughout the world (1–3). Chickens are natural hosts for *C. jejuni* and serve as a model system for analyzing commensalism by *C. jejuni* (4–6). In these avian hosts, *C. jejuni* predominantly colonizes the mucous layer and crypts of the lower intestinal tract, including the ceca and large intestine, with much lower abundance in the upper intestinal tract (i.e., small intestine) (4, 5). In humans, *C. jejuni* also infects the lower intestinal tract, including the colon and rectum. The ability of *C. jejuni* to adhere to and invade the colonic epithelium contributes to the pathogenesis of inflammatory diarrheal disease (7). How *C. jejuni* differentiates between regions of the intestinal tract to locate preferred niches to establish either a persistent, asymptomatic colonization in avian species or a productive infection in humans is largely unknown.

Compared to many other enteric pathogens, *C. jejuni* possesses a very limited carbohydrate catabolism due to lack of enzymes to utilize many sugars as a carbon source (8, 9). Catabolism of glucose occurs in some *Campylobacter coli* isolates, but only one subspecies of *C. jejuni* has been found to produce a system for the uptake and utilization of this carbohydrate (10). A subset of *C. jejuni* strains possesses a genomic island encoding enzymes for fucose utilization and a chemotaxis receptor for fucose (11–13). Instead, most *C. jejuni* strains predominantly rely on amino acids and peptides to fuel various metabolic pathways, including the tricarboxylic acid (TCA) cycle and gluconeogenesis for lipo-oligosaccharide and capsular polysaccharide biogenesis (8, 9). Various studies have revealed that serine, aspartate, glutamate, and proline are used preferentially by *C. jejuni* to support *in vitro* growth (14–16). As such, *C. jejuni* strains also produce specific transporters for these amino acids (16–19). Additionally, a subset of *C. jejuni* strains have an expanded metabolic repertoire for utilization of asparagine and glutamine for growth (15). Ultimately, these amino acids are catabolized into various carbon sources that feed the TCA cycle during normal microaerobic growth conditions (10% CO_2_, 5% O_2_, and 85% N_2_) for the bacterium. *C. jejuni* produces all components of a full TCA cycle and runs in the oxidative direction under microaerobic conditions (20). Oxygen is the favored electron acceptor in microaerobic conditions for maximal ATP generation. A key enzyme in the *C. jejuni* TCA cycle is the bifunctional FrdABC complex, which serves as a succinate dehydrogenase under microaerobic conditions to maintain the TCA cycle running in the oxidative direction, and as a fumarate reductase to convert fumarate to succinate, which is secreted, under strict oxygen-limited conditions *in vitro* (i.e., minimal O_2_ well below 5%) (16, 20, 21). Under these oxygen-limited conditions, alternative electron acceptors such as formate, nitrate, and nitrite can be used (22, 23).

**FIG 1:**
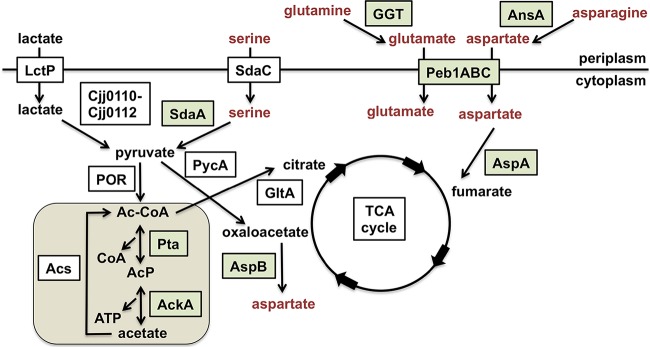
Metabolic reactions in *C. jejuni* containing select amino acid transport and catabolic pathways. A simplified schematic of the *C. jejuni* amino acid transport and catabolic pathways whose expression was affected by the acetogenesis pathway as presented in this work is shown. Amino acids in red indicate the amino acids that are preferred by *C. jejuni* for optimal *in vivo* or *in vitro* growth. Proteins in boxes are those required for the associated processes. Proteins in green are shown in this work or have been previously shown to be required for commensal colonization of the avian intestinal tract or infection of the murine intestinal tract. The acetogenesis pathway of *C. jejuni* is shown on tan background.

In addition to amino acids, *C. jejuni* catabolizes keto acids, organic acids, and short-chain fatty acids (SCFAs) such as pyruvate, lactate, and acetate (Fig. 1). Exogenously acquired l-lactate is oxidized to pyruvate by one of two different enzymes in *C. jejuni* (24). In addition, serine and phosphoenolpyruvate are substrates to generate pyruvate (18, 25, 26). Once pyruvate is available, *C. jejuni* can convert pyruvate to acetate by the multistep acetogenesis pathway (14). In this pathway, pyruvate is first converted to acetyl coenzyme A (Ac-CoA) via pyruvate oxidoreductase (POR), Ac-CoA is converted to acetyl-phosphate (Ac-P) via phosphotransacetylase (Pta), and Ac-P is dissimilated to acetate and ATP via acetate kinase (AckA) (extensively reviewed in references 14 and 27). As in many bacteria, acetogenesis during exponential growth results in secretion of acetate through an unknown transporter of *C. jejuni* (14, 27). However, depletion of carbon sources in stationary phase causes a metabolic “acetate switch” in many bacteria, where acetate is imported and converted by Ac-CoA synthase (Acs) to Ac-CoA, which can be used to fuel the TCA cycle (27). In addition, the acetogenesis pathway also produces Ac-P as an intermediate, which we have shown can impact a signal transduction pathway by serving as phosphodonor to a response regulator and affect gene expression in *C. jejuni* (28). As such, the acetogenesis pathway may impact both metabolic and signal transduction pathways in a bacterial cell.

In a previous study to identify genes of *C. jejuni* required for infection of newly hatched chicks, we identified a mutant with a transposon insertion in *pta* with a 10-fold defect in commensal colonization of the ceca after 7 days of infection (5). Since this mutation eliminates a central enzyme of the acetogenesis pathway, we explored the reason why a *C. jejuni* acetogenesis mutant is attenuated for commensal colonization of the natural avian host. Our discoveries suggest that *C. jejuni* possesses a mechanism for recognizing the spatial distribution of SCFAs and lactate in the avian intestinal tract as a potential marker to identify preferred niches for optimal commensal colonization. Furthermore, the acetate and butyrate SCFAs that are abundant in the lower intestinal tracts in chicks (29; reviewed in reference 30) are likely *in vivo* cues recognized by *C. jejuni* to activate expression of a specific set of genes encoding catabolic enzymes and transport systems for amino acids and other metabolic enzymes known to be required for *in vivo* growth. In contrast, lactate, which is abundant in the upper intestinal tract where *C. jejuni* less efficiently colonizes, repressed expression of these same genes. We propose that the spatial distribution of these metabolites in the chick intestinal tract allows *C. jejuni* to discriminate between different regions of the intestines and coordinate expression of determinants necessary for commensal colonization of the ceca and large intestines. Our findings are also likely relevant for cues that *C. jejuni* may recognize in the human intestinal tract to promote infection and diarrheal disease.

## RESULTS

### The acetogenesis pathway is required for initial commensal colonization of the avian intestinal tract.

*C. jejuni* produces an acetogenesis pathway with Pta to convert Ac-CoA to Ac-P (and release CoA) and AckA to convert Ac-P to ATP and acetate (Fig. 1) (14, 27). Our previous study revealed that upon infecting chicks on the day they hatched, a *C. jejuni* mutant with a Tn insertion in *pta* exhibited attenuated commensal colonization of the chick ceca on day 7 postinfection (5). The arrangement of *pta* and *ackA* on the *C. jejuni* genome suggests that the genes could form a cotranscribed operon. Thus, the *C. jejuni pta*::Tn mutant may also fail to transcribe the downstream *ackA* gene. Indeed, we observed that transcription of *ackA* was greatly reduced in the *pta*::Tn mutant compared to wild-type (WT) *C. jejuni* (data not shown).

**FIG 2:**
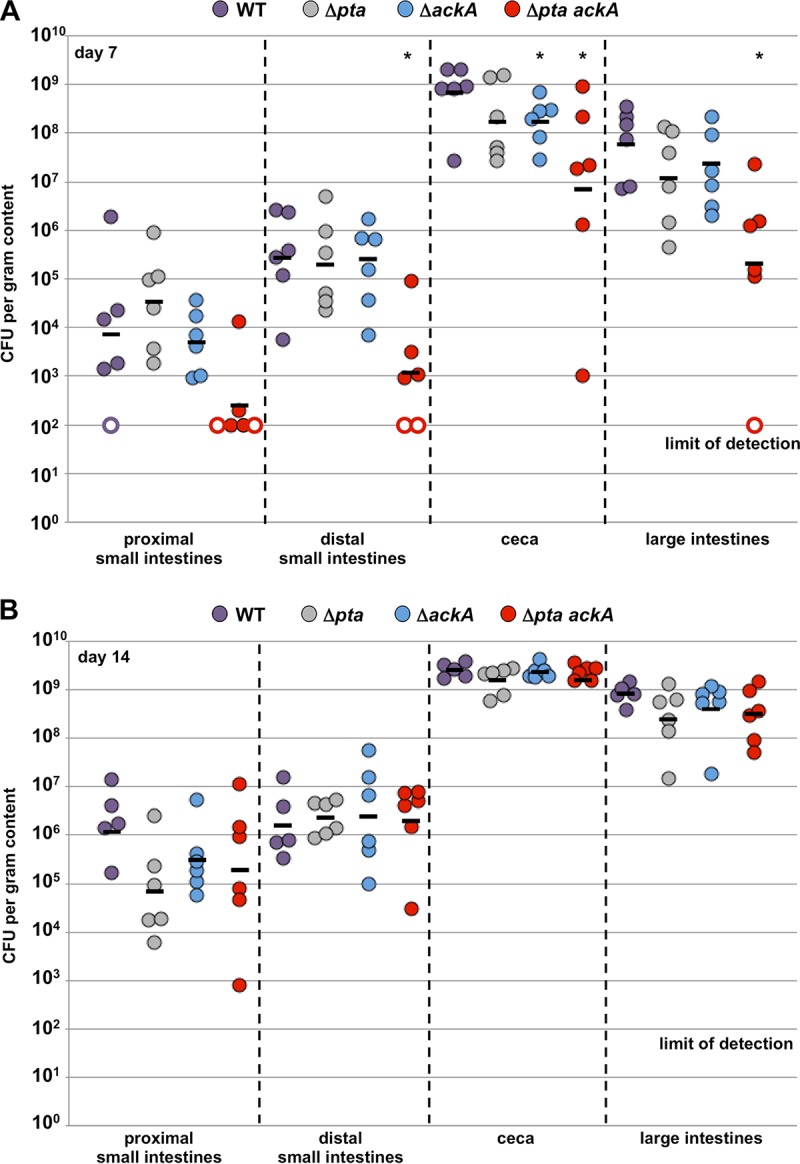
Colonization dynamics of WT *C. jejuni* and isogenic acetogenesis mutants over time in the avian intestinal tract. On the day the chicks hatched, the chicks were orally infected with approximately 100 CFU of WT *C. jejuni* 81-176 Sm^r^ or isogenic Δ*pta*, Δ*ackA*, or Δ*pta ackA* mutants with different defects in the *C. jejuni* acetogenesis pathway. Chicks were sacrificed on day 7 (A) or day 14 (B) postinfection, and the level of each *C. jejuni* strain in the proximal small intestine, distal small intestine, ceca, and large intestine was determined (reported as CFU per gram of content). Each closed circle represents the level of *C. jejuni* in a single chick. Open circles represent chicks with *C. jejuni* levels below the limit of detection (<100 CFU per gram of content). Horizontal bars represent the geometric means for the groups. Statistical analysis was performed using the Mann-Whitney *U* test. Values that were significantly different (*P* < 0.05) from the WT value are indicated by an asterisk.

We constructed new mutants from *C. jejuni* 81-176 Sm^r^ Δ*astA*. This mutant lacks AstA, an arylsulfatase enzyme that is not required for chick infection and has been used as a transcriptional reporter in our previous studies (31–33); this strain is considered the WT strain for all analyses in this report. We deleted *pta*, *ackA*, or both genes from the WT chromosome and then analyzed which components of the acetogenesis pathway were required for commensal colonization of the ceca of chicks on the day they hatched. We confirmed that *ackA* was expressed at WT levels in the Δ*pta* mutant, which is in contrast to the original *pta*::Tn mutant (data not shown). We then compared the commensal colonization capacity of these mutants with WT *C. jejuni*. After oral inoculation of chicks with approximately 10^2^ CFU on the day they hatched, the WT strain colonized throughout the avian intestinal tract on days 7 and 14 postinfection, with the highest levels in the ceca and large intestines (10^8^ to 10^9^ CFU per g of content) and lower levels in the proximal and distal small intestines (10^4^ to 10^6^ CFU per g of content) (Fig. 2A and B). The levels of colonization of either the Δ*pta* or Δ*ackA* mutant in different regions of the intestinal tract were comparable to those of the WT on days 7 and 14 postinfection (Fig. 2A and B). However, we did observe a fourfold reduction in cecal colonization on day 7 postinfection by these mutants that was statistically significant only for the Δ*ackA* mutant. In addition, these mutants were reduced 2.5- to 5-fold in the large intestines at day 7 postinfection, but this difference did not meet statistical significance.

In contrast, *C. jejuni* Δ*pta ackA* displayed a large colonization defect throughout the intestinal tract on day 7 postinfection (Fig. 2A). We observed 29- and 229-fold reductions in colonization of the proximal and distal small intestines, respectively, with no *C. jejuni* detected in these regions in two chicks. In the lower intestinal tract, we observed 98- and 294-fold decreases in colonization of the ceca and large intestines, respectively, by the Δ*pta ackA* mutant (Fig. 2A). The level of *C. jejuni* Δ*pta ackA* in the large intestines was below the limit of detection in one chick. The colonization defect of this Δ*pta ackA* mutant is consistent with that of the original isolated *pta*::Tn mutant that lacks expression of both *pta* and *ackA* (5). However, the Δ*pta ackA* mutant colonized all intestinal regions at close to WT levels when the infection period was extended to day 14 postinfection (Fig. 2B).

### Physiological defects of the *C. jejuni* acetogenesis mutant.

We next explored why the *C. jejuni* Δ*pta ackA* mutant had a significant commensal colonization defect for the avian intestinal tract. As described above, the acetogenesis pathway is disrupted in this mutant, which may cause the accumulation of Ac-CoA and the reduction of both Ac-P and acetate (Fig. 1). To assess the production of acetate in *C. jejuni* strains, we inoculated *C. jejuni* into *Campylobacter* defined medium (CDM) and monitored production of acetate during growth of the WT strain and acetogenesis mutant for up to 32 h. CDM contains all amino acids (with most amino acids around 1 mM and serine the most abundant at 19 mM) and the keto acids pyruvate and α-ketoglutarate (5 to 9 mM), which together are the primary carbon sources for *C. jejuni* during growth in this medium (34). The WT strain produced up to 50 mM acetate and consistently secreted 2.5- to 20-fold more acetate than the Δ*pta ackA* mutant did (Table 1). We were unable to obtain an accurate quantification of Ac-P due to the inherent instability of the metabolite.

**TABLE 1:** Acetate production by WT *C. jejuni* and the Δ*pta ackA* acetogenesis mutant

Time (h)	Acetate concn (mM)^a^	
	Wild-type	Δ*pta ackA* mutant
4	16.5 ± 3.1	<0.8 ± 1.3*
8	35.5 ± 4.8	5.2 ± 0.2*
12	50.5 ± 6.5	11.2 ± 2.0*
16	44.1 ± 1.3	12.1 ± 1.9*
20	50.4 ± 1.3	20.3 ± 1.5*
24	37.4 ± 3.3	18.3 ± 1.3*
28	32.6 ± 3.2	15.9 ± 0.9*
32	30.8 ± 3.3	17.8 ± 3.3*

^a^ *C*. *jejuni* strains were inoculated in CDM broth at starting OD_600_ of 0.1. The concentration of acetate was measured in culture-free supernatants in triplicate at indicated times after growth, and all samples were normalized to an OD_600_ of 1.0. Values that are significantly different (*P* < 0.05) from the value for the wild type by Student’s two-tailed *t* test are indicated by an asterisk.

**FIG 3:**
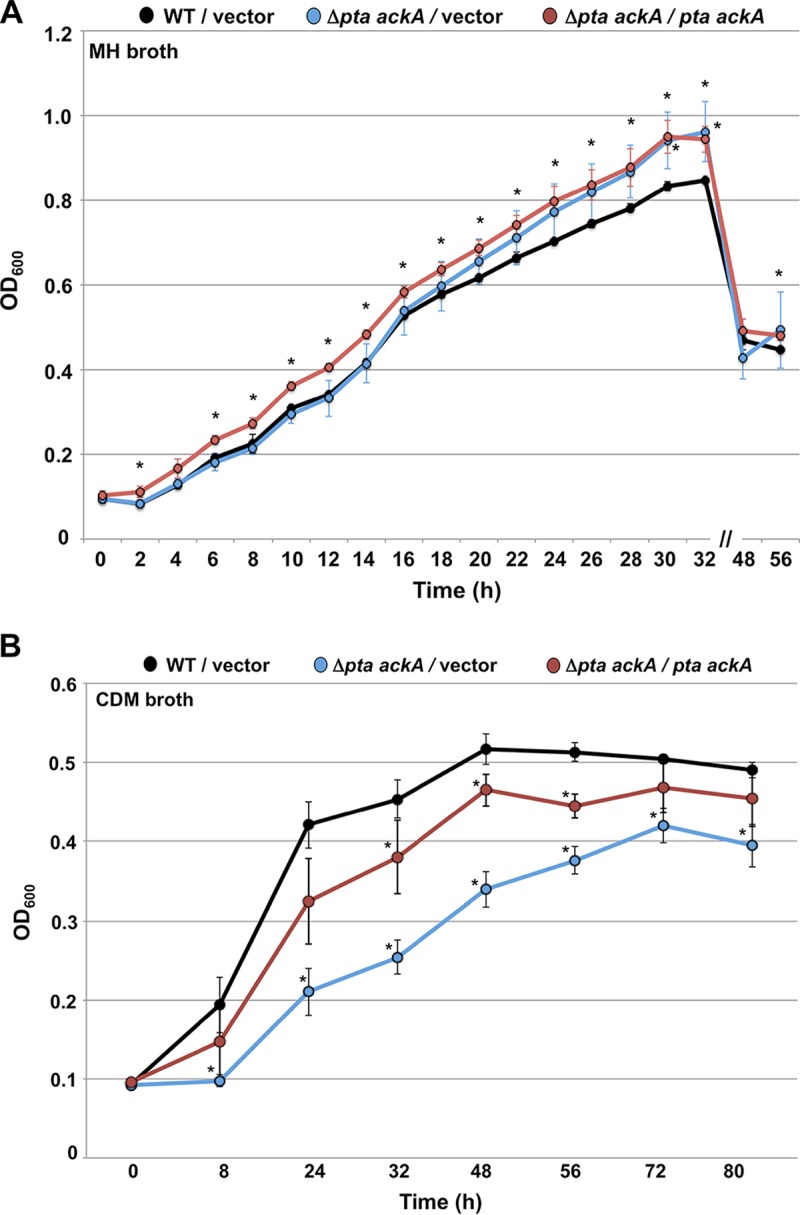
Analysis of growth of C. jejuni strains in rich and defined media. *C. jejuni* strains were grown in Mueller-Hinton (MH) broth (A) or *Campylobacter* defined medium (CDM) broth (B) in microaerobic conditions at 37°C for 56 to 80 h. Strains used for analysis include WT *C. jejuni* 81-176 Sm^r^ containing pDAR1423 (empty vector) (black circles and line), 81-176 Sm^r^ Δ*pta ackA* containing pDAR1423 (empty vector) (blue circles and line), and 81-176 Sm^r^ Δ*pta ackA* containing pPML1071 (*pta ackA* in pDAR1423) (red circles and line). Strains were analyzed in triplicate, and the data are presented as an average of OD_600_ readings for strains at each time point. Error bars indicate standard deviations. Values for mutants that are statistically significantly different (*P* < 0.05) from the value for the WT strain are indicated by an asterisk.

The lack of Pta and AckA likely causes accumulation of Ac-CoA that cannot enter the acetogenesis pathway and must be converted to citrate (Fig. 1). This mutant may also have an alteration in the levels of free CoA as a consequence. CoA is an essential cofactor for enzymes in many metabolic and physiological pathways. Thus, if levels of free CoA are reduced in *C. jejuni* Δ*pta ackA*, the mutant may have both an *in vivo* and *in vitro* growth defect. However, the Δ*pta ackA* mutant grew to levels similar to those of the WT and grew slightly better at late exponential phase in complex Mueller-Hinton (MH) broth (Fig. 3A). Complementation of the acetogenesis mutant with *pta* and *ackA* modestly enhanced growth over the WT strain. WT *C. jejuni* grew slower in CDM than in MH broth over the course of the assay (Fig. 3B). In comparison, the Δ*pta ackA* mutant showed a significant reduction in growth relative to WT *C. jejuni* in the defined medium (Fig. 3B). Complementation of the mutant with a plasmid with genes with the ability to express both *pta* and *ackA* in *trans* largely restored growth of the mutant to WT levels in CDM. Thus, the acetogenesis mutant has a defect in growth under conditions where keto and amino acids are primary carbon sources.

### Transcriptional defects of *C. jejuni* Δ*pta ackA*.

As discussed above, we found that the Δ*pta ackA* acetogenesis mutant produced reduced levels of acetate. Additionally, this mutant would be predicted to have a reduction in Ac-P, an intermediate in the acetogenesis pathway. Both acetate and Ac-P can influence signal transduction pathways as a stimulus (acetate), phosphodonor (Ac-P), or modification substrate (Ac-P) and impact transcription of genes in bacteria (35–41). Therefore, we examined whether *C. jejuni* Δ*pta ackA* might have an altered transcription profile that could contribute to its attenuated commensal colonization phenotype. To this end, we grew WT *C. jejuni* and the Δ*pta ackA* mutant in MH broth in microaerobic conditions at 37°C to mid-log phase and then isolated mRNA for transcriptome analysis using DNA microarrays. A select list of genes whose expression was increased or decreased by at least twofold in the Δ*pta ackA* mutant are reported in Table 2 (a full list of genes whose expression was altered is shown in Table S1 in the supplemental material).

10.1128/mBio.00407-17.3 TABLE S1  Complete list of genes differentially expressed in *C. jejuni* 81-176 Sm^r^ Δ*pta ackA* compared to WT *C. jejuni* 81-176 Sm^r^. Download TABLE S1, PDF file, 0.1 MB. Copyright © 2017 Luethy et al. 2017 Luethy et al. This content is distributed under the terms of the Creative Commons Attribution 4.0 International license.

**TABLE 2:** Condensed list of genes differentially expressed in *C. jejuni* 81-176 Sm^r^ Δ*pta ackA* compared to WT *C. jejuni* 81-176 Sm^r^ by microarray analysis^a^

Locus tag	Gene	Putative function(s)	WT/Δ*pta ackA* ratio^b^
*Cjj81176_0038*	*rrc*	Rbo/Rbr-like protein of *C. jejuni*; rubrerythrin-like protein	9.50
*Cjj81176_0056*	*ansA*	l-Asparginase	7.28
*Cjj81176_0067*	*ggt*	γ-Glutamyl transferase	8.11
*Cjj81176_0122*	*aspA*	Aspartate ammonia lyase	7.51
*Cjj81176_0123*	*dcuA*	Anaerobic C_4_-dicarboxylate transporter	5.22
*Cjj81176_0124*	*dcuB*	Anaerobic C_4_-dicarboxylate transporter	6.19
*Cjj81176_0682*		Possible di-/tripeptide transporter	2.61
*Cjj81176_0683*		Possible di-/tripeptide transporter	5.90
*Cjj81176_0927*	*peb1a*	Aspartate/glutamate transporter, permease component	2.74
*Cjj81176_0928*	*peb1b*	Aspartate/glutamate transporter, solute-binding component	5.48
*Cjj81176_0929*	*peb1c*	Amino acid transporter, ATP-binding component	5.11
*Cjj81176_1615*	*sdaA*	l-Serine dehydratase	2.66
*Cjj81176_1616*	*sdaC*	l-Serine transporter	2.75
*Cjj81176_0315*	*peb3*	Glycoprotein; putative adhesion or transport protein	0.22

^a^ A subset of genes identified to be differentially expressed in mutants that were further analyzed in this work is shown here. A complete list of genes that were differentially expressed in mutants is shown in Table S1 in the supplemental material.

^b^ Expression of genes was increased or decreased by twofold in the *C. jejuni* Δ*pta ackA* mutant compared to the WT *C*. *jejuni*.

**FIG 4:**
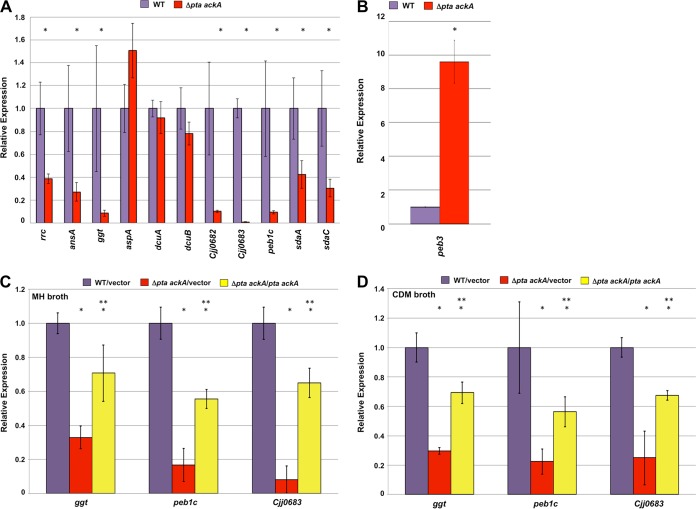
Transcriptional analysis of acetogenesis-dependent genes of *C. jejuni* (A and B). Semiquantitative real-time PCR analysis of transcription of a subset of genes initially identified by microarray analysis of WT *C. jejuni* and *C. jejuni* Δ*pta ackA*. The level of expression of each gene in the WT *C. jejuni* 81-176 as measured by qRT-PCR was set at 1. Expression of each gene in the Δ*pta ackA* mutant is shown relative to the WT strain. (A) Genes whose expression was reduced in the Δ*pta ackA* mutant relative to WT *C. jejuni* in the microarray analysis. (B) Gene whose expression was increased in the Δ*pta ackA* mutant relative to WT *C. jejuni* in the microarray analysis. (C and D) Semiquantitative real-time PCR analysis of transcription of select members of the SCFA-induced genes of *C. jejuni* in different media in WT *C. jejuni* and in the Δ*pta ackA* mutant with or without complementation in *trans*. The expression of *ggt*, *peb1c*, and *Cjj0683* in WT *C. jejuni* 81-176 Sm^r^ Δ*astA* with empty vector pDAR1423 (purple) as measured by qRT-PCR was set at 1. Expression of each gene in Δ*pta ackA* with empty vector pDAR1423 (red) or Δ*pta ackA* with vector pPML1071 containing WT *pta ackA* (yellow) is shown relative to the WT strain. Strains were examined in triplicate after growth in MH broth (A to C) or in CDM broth (D). Error bars indicate standard deviations. Statistically significant differences in gene expression between WT *C. jejuni* and mutant strains (*, *P* < 0.05) or between Δ*pta ackA* mutant with vector alone or the Δ*pta ackA* mutant complemented with WT *pta ackA* (**, *P* < 0.05) as performed by the Student’s *t* test are indicated.

Many genes with decreased expression in the *C. jejuni* Δ*pta ackA* acetogenesis mutant encode components of the amino acid transport or catabolism system (Fig. 1), some of which have previously been shown to be required for optimal colonization of avian or murine hosts. Additionally, we noted that transcription of a number of genes affecting energy metabolism were decreased in the acetogenesis mutant, although generally not as severely as amino acid transport and catabolism systems. We suspect that some of these transcriptional defects in amino acid utilization may contribute to the colonization defect in chicks and the growth defect in CDM broth we observed (Fig. 2A and 3B). For example, expression of *ggt*, *ansA* (also referred to as *ansB*), *peb1c*, *sdaA*, and *sdaC*, which encode factors for acquisition or utilization of glutamine, glutamate, serine, asparagine, and aspartate, were 2.4- to 11.6-fold lower in the acetogenesis mutant when assessed by semiquantitative real-time PCR (qRT-PCR) (Fig. 4A). GGT is a γ-glutamyltransferase that converts glutamine or glutathione to glutamate (42). Cleavage of glutathione by GGT in *C. jejuni* in conjunction with another peptidase also supplies the bacterium with cysteine (43). The ability of GGT to generate glutamate is required by *C. jejuni* for persistent commensal colonization of the chick ceca and infection of the intestinal tracts and livers of *myd88*^−*/*−^ mice (15, 44, 45). AnsA is an l-asparaginase that converts asparagine to aspartate and is required for growth in the livers of *myd88*^−*/*−^ mice upon intraperitoneal infection (15). Peb1c is the ATPase component of a *C. jejuni* amino acid transport system with Peb1a and Peb1b that is specific for the acquisition of aspartate, glutamate, and glutamine (17). SdaC is required for serine transport, and SdaA is a l-serine dehydratase that converts serine to pyruvate, which not only is converted to Ac-CoA to feed the acetogenesis pathway but can also be converted to oxaloacetate to feed the TCA cycle (18). A previous study found that Peb1A of the Peb1 system and SdaA are required for WT levels of commensal colonization of chicks and for growth in the intestines and livers of WT, *myd88*^−*/*−^, or *nramp1*^−*/*−^ mice (18, 46–48).

We also noted that expression of *Cjj81176_0683* (*Cjj0683*) and *Cjj81176_0682* (*Cjj0682*) was reduced 112.4- and 9.7-fold, respectively (Fig. 4A). Not all *C. jejuni* strains have a locus containing these genes. When this locus is present in *C. jejuni*, it contains only one gene that encodes a large protein that is predicted to function as a di- or tripeptide permease of a transport system, although its biological activity has not been evaluated (43). In *C. jejuni* 81-176, a mutation has occurred that results in two separate open reading frames, *Cjj0683* and *Cjj0682*. It is unknown whether the putative encoded proteins produce a functional permease. We also noted that *rrc* transcription was reduced 2.6-fold in the acetogenesis mutant. This gene is hypothesized to encode a desulforubrerythrin that may be involved in resistance to oxidative stress (49).

Although the microarray analysis suggested that transcription of *aspA*, *dcuA*, and *dcuB* was significantly reduced, we were unable to verify a decrease in transcription by qRT-PCR (Fig. 4A). In a previous study, these genes are responsible for the uptake of aspartate and conversion of aspartate to fumarate (16). AspA had previously been shown to be required for intracellular survival in human colonic cells, infection of *myd88*^−*/*−^ *nramp1*^−*/*−^ mice, and commensal colonization of chicks (16, 50).

Other previously identified commensal colonization determinants whose expression was reduced according to microarray analysis include *Cjj81176_0382*, *fedB*, *ciaI*, and *cadF* (Table S1) (51–54). In addition, *C. jejuni* Δ*ciaI* had an approximately twofold reduction in invasion in human colonic cells (51, 52, 55). Microarray analysis also revealed a reduction in expression of *frdB* and *frdC*, which with *frdA*, encode the FrdABC complex with succinate dehydrogenase and fumarate reductase activity (56). FrdA is required for WT levels of cecal colonization of chicks (56). For other *C. jejuni* determinants required for invasion or intracellular survival in human colonic cells, we noted a reduction in expression of *aspB* and *ciaC* by microarray analysis (Table S1). AspB is required to synthesize aspartate from oxaloacetate and glutamate and for infection of *myd88*^−/−^ *nramp1*^−/−^ mice (Fig. 1) (50). The exact function of CiaC in invasion of host cells is unclear (57). Other notable genes whose expression was reduced include *mfrABC* and *Cjj0291-Cjj0292*, which are required for fumarate reduction and trimethylamine-*N*-oxide (TMAO) or dimethyl sulfoxide (DMSO) reduction, respectively (20, 22). These systems may be important for energy production during host colonization.

We did find that expression of a smaller number of genes appeared to be increased in *C. jejuni* Δ*pta ackA* relative to WT *C. jejuni* (Table 2 and Table S1). We verified that expression of one gene, *peb3*, was increased 10-fold (Fig. 4B). Peb3 is a surface-localized glycoprotein that may function as an adhesin for attachment to eukaryotic cells or a transport protein for phosphate-containing compounds (58–61).

For all analyses reported in the remainder of this work, we analyzed transcription of *ggt*, *peb1c*, and *Cjj0683* as representatives of a set of genes affected by the acetogenesis pathway. Upon complementation of the Δ*pta ackA* mutant with a vector or plasmid with genes with the ability to express *pta* and *ackA* in *trans*, we noted that expression of these genes during growth in MH broth was significantly higher than the mutant harboring vector alone, although the levels of expression were not completely restored to WT levels (Fig. 4C).

We also assessed whether transcription of these genes was reduced in CDM broth, which may contribute to the growth defect of the Δ*pta ackA* mutant in this medium (Fig. 3B). Compared to the WT *C. jejuni*, the Δ*pta ackA* mutant showed four- to fivefold reductions in expression of *ggt*, *peb1c*, and *Cjj0683* (Fig. 4D). Upon complementation of the Δ*pta ackA* mutant with *pta* and *ackA*, expression of these genes was partially restored to WT levels and was significantly higher than in the Δ*pta ackA* mutant with vector alone. Thus, the acetogenesis pathway of *C. jejuni* influences expression of these genes.

### Colonization capacity of Δ*ggt*, Δ*peb1c*, and Δ*Cjj0683* mutants.

**FIG 5:**
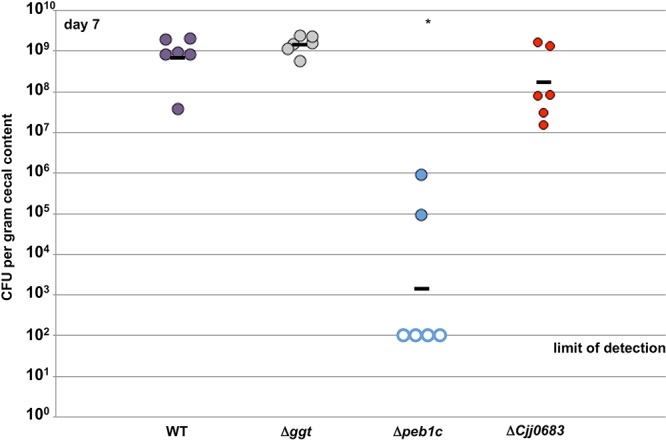
Cecal colonization capacities of WT *C. jejuni* and isogenic mutants lacking a single select gene of acetogenesis-dependent genes for the avian intestinal tract. On the day the chicks hatched, they were orally infected with approximately 100 CFU of WT *C. jejuni* 81-176 Sm^r^ or isogenic Δ*ggt*, Δ*peb1c*, or Δ*Cjj0683* mutants. The chicks were sacrificed on day 7 postinfection, and the level of each *C. jejuni* strain in ceca was determined (reported as CFU per gram of cecal content). Each closed circle represents the level of *C. jejuni* in a single chick. Open circles represent chicks with *C. jejuni* levels below the limit of detection (<100 CFU per gram of content). Horizontal bars represent geometric means for the groups. Statistical analysis was performed using the Mann-Whitney *U* test (*, *P* < 0.05).

We next investigated whether genes dependent upon the acetogenesis pathway for WT levels of transcription are required for initial commensal colonization of the chick ceca. We constructed *C. jejuni* mutants lacking *ggt*, *peb1c*, or *Cjj0683* and orally gavaged chicks with approximately 10^2^ CFU on the day they hatched. On day 7 postinfection, WT *C. jejuni* colonized the chicks at around 10^9^ CFU per gram of cecal content (Fig. 5). We observed similar levels of colonization by the Δ*ggt* and Δ*Cjj0683* mutants. The lack of a colonization defect by our Δ*ggt* mutant on day 7 postinfection is consistent with another study that found that GGT was required for persistent colonization of chicks on day 35 postinfection, but not at an earlier time point of 5 days postinfection (44). In contrast, *C. jejuni* Δ*peb1c* demonstrated a drastic reduction in cecal colonization of more than 100,000-fold with the bacterium not detected in four of the six chicks. This colonization defect is similar to previous studies demonstrating that *peb1a* was required by *C. jejuni* for commensal colonization of chicks and growth in the murine host (46–48).

### Exogenous acetate restores expression of genes to a *C. jejuni* acetogenesis mutant.

**FIG 6:**
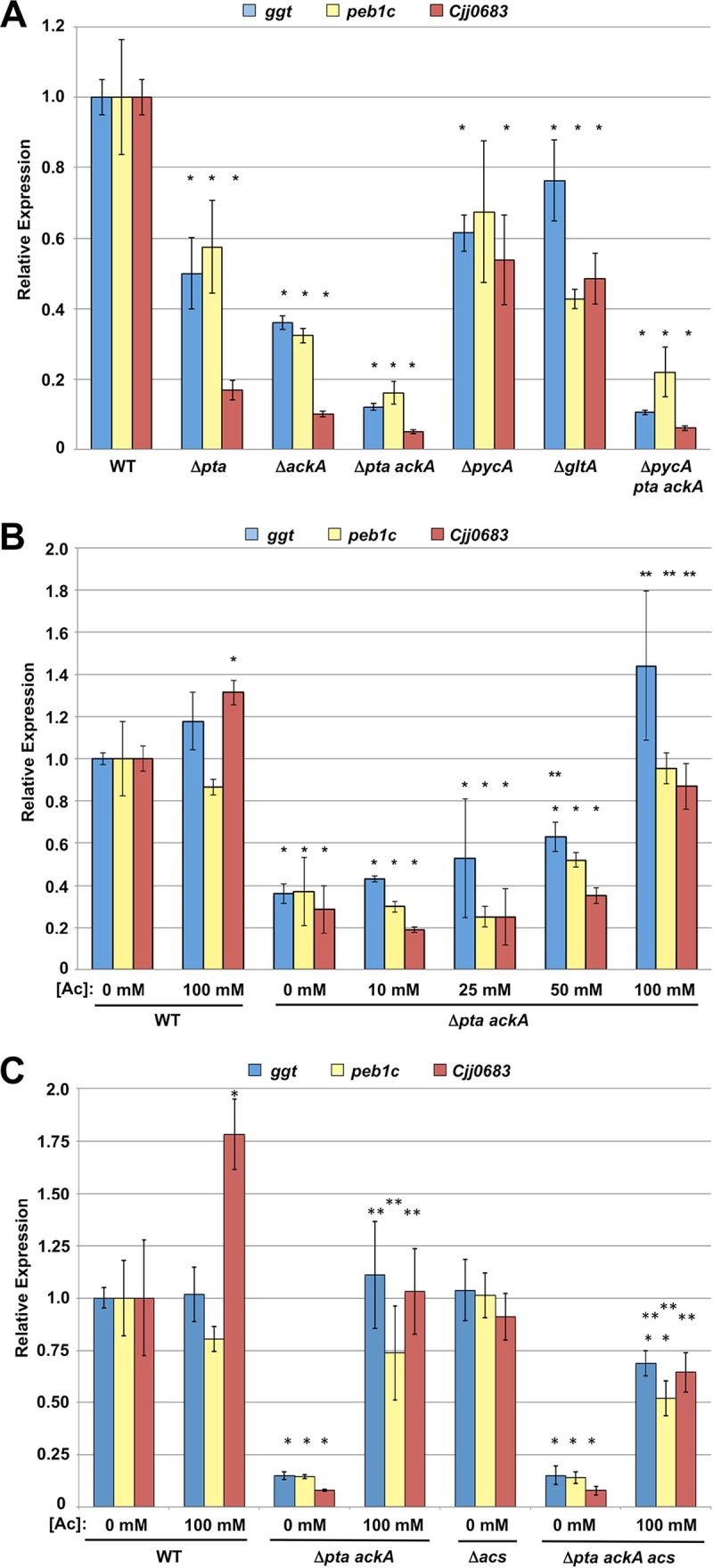
Expression of the acetogenesis-dependent genes in WT and isogenic acetogenesis mutants upon exogenous acetate supplementation. (A to C) Semiquantitative real-time PCR analysis of transcription of *ggt*, *peb1c*, and *Cjj0683* in WT *C. jejuni* and isogenic mutants grown in CDM (A) or CDM with different concentration of exogenous acetate (Ac) (B and C). All media were equilibrated to pH 7.0 prior to the growth of bacteria to eliminate effects due to acidification by exogenous acetate. The levels of expression of *ggt*, *peb1c*, and *Cjj0683* in the WT *C. jejuni* 81-176 grown without acetate as measured by qRT-PCR were set at 1. Expression of each gene in the WT strain grown with acetate or mutants grown with or without acetate is shown relative to the WT strain grown without acetate. Error bars indicate standard deviations. Statistically significant differences in gene expression between WT *C. jejuni* without acetate and strains with or without acetate (*, *P* < 0.05) or between each individual strain without acetate or with acetate (**, *P* < 0.05) performed by the Student’s *t* test are indicated.

The decreased expression of colonization genes in the *C. jejuni* Δ*pta ackA* mutant might be due to a physiological effect that mutation of the acetogenesis pathway has on the bacterium such as reduced acetate or Ac-P or increased Ac-CoA that may cause fluctuations in intermediates for the TCA cycle. To narrow down the possibilities for how the acetogenesis pathway may be affecting expression of colonization genes, we analyzed single Δ*pta* or Δ*ackA* mutants to determine how changes in Ac-CoA, Ac-P, or acetate levels may impact expression of the acetogenesis-dependent genes. Deletion of Δ*pta* or Δ*ackA* caused around 2- to 10-fold reductions in expression of *ggt*, *peb1c*, and *Cjj0693* (Fig. 6A). Although these levels were significantly lower than the levels produced by the WT, the Δ*pta ackA* mutant was more severely defective for expression of all of these genes. The Δ*pta* mutant would be expected to produce reduced levels of Ac-P, and the Δ*ackA* mutant would be expected to accumulate Ac-P. Previous analyses of Ac-P-dependent gene expression of flagellar genes correlate with these relative higher and lower values of Ac-P in *C. jejuni pta* and *ackA* mutants (28). However, these mutants showed reductions in expression of *ggt*, *peb1c*, and *Cjj0683*, regardless of having higher or lower levels of Ac-P. Thus, we concluded that the expected reduction of Ac-P in the Δ*pta ackA* mutant is likely not the cause of the reduced expression of acetogenesis-dependent genes in this mutant.

Since the Δ*pta*, Δ*ackA*, or Δ*pta ackA* mutations might result in increases in Ac-CoA and alterations in the levels of substrates provided to the TCA cycle, we analyzed expression of these colonization genes in *C. jejuni* Δ*pycA* or Δ*gltA* mutants. *C. jejuni* Δ*pycA* lacks the enzyme to convert pyruvate to oxaloacetate, whereas *C. jejuni* Δ*gltA* lacks the enzyme to convert Ac-CoA to citrate for the TCA cycle (Fig. 1). Both mutations are likely to cause an increase in Ac-CoA. In the Δ*pycA* mutant, increased Ac-CoA may be shunted toward the acetogenesis pathway or the TCA cycle by its conversion to citrate by GltA. In the Δ*gltA* mutant, accumulated Ac-CoA would likely be shunted to the acetogenesis pathway, with reduced citrate generated for the TCA cycle. The levels of expression of the acetogenesis-dependent genes in these mutants were reduced only 2.5-fold or less and were not as low as the levels of most of the colonization genes in the Δ*pta*, Δ*ackA*, or Δ*pta ackA* mutants (Fig. 6A). Furthermore, deletion of *pycA* in the Δ*pta ackA* mutant phenocopied identically the Δ*pta ackA* mutant (Fig. 6A). We attempted to construct and analyze a Δ*pta ackA gltA* mutant, but deletion of *gltA* in the Δ*pta ackA* background is apparently lethal. These findings lend greater support that a specific effect of the acetogenesis pathway, rather than altered levels of Ac-CoA or substrates to the TCA cycle, impact expression of colonization genes.

We next analyzed whether reduced acetate generation of the *C. jejuni* Δ*pta ackA* mutant was the cause of the deficient expression of acetogenesis-dependent genes. To this end, we monitored expression of *ggt*, *peb1c*, and *Cjj0683* in the WT and in the Δ*pta ackA* mutant after exposure to a range of acetate concentrations. We incubated *C. jejuni* strains in CDM broth rather than MH broth to finely control the composition of the medium. For WT *C. jejuni*, addition of 100 mM acetate did not significantly influence expression of *ggt* or *peb1c* and only modestly increased expression of *Cjj0683* (Fig. 6B). However, a dose-dependent increase in gene expression in *C. jejuni* Δ*pta ackA* occurred with all genes by acetate supplementation. Increases in gene expression occurred with 50 mM acetate, and the levels of expression of the genes in the Δ*pta ackA* mutant were fully restored to WT levels with 100 mM acetate (Fig. 6B).

We postulated that the stimulatory effect of acetate on gene expression may be due to dissimilation of acetate in a catabolic pathway or as a direct cue recognized by *C. jejuni* to specifically influence transcription of these genes. However, the stimulatory effect was observed in a *C. jejuni* Δ*pta ackA* mutant, which disrupts the only known and reversible enzymes for the production of Ac-P and Ac-CoA from acetate (Fig. 1). Currently, the only other known utilization pathway for acetate in *C. jejuni* is the direct conversion of acetate to Ac-CoA via Acs that occurs during the “acetate switch” many bacteria experience in late phases of growth (14, 27). We tested the potential for Acs to convert acetate to Ac-CoA for the acetate-dependent effects on expression of *ggt*, *peb1c*, and *Cjj0683* by deleting *acs* from WT *C. jejuni* and *C. jejuni* Δ*pta ackA*. These genes were expressed at WT levels in *C. jejuni* Δacs and at a level similar to the reduced level of Δ*pta ackA* in the Δ*pta ackA acs* mutant (Fig. 6C). Upon addition of 100 mM acetate to the Δ*pta ackA acs* mutant, we found a 4- to 8-fold increase in gene expression that was just ~25% lower than in the WT strain without acetate or in the Δ*pta ackA* mutant with 100 mM acetate (Fig. 6C). We interpret these data as demonstrating that acetate conversion to Ac-CoA by Acs accounts for only a small amount of the gene expression restored upon the addition of acetate to the acetogenesis mutant. Thus, a *C. jejuni* mutant lacking all known acetate catabolism pathways still restored most of the expression of the acetogenesis-dependent genes upon acetate supplementation with acetate. These findings suggest that acetate is a cue recognized by *C. jejuni* to stimulate gene expression; however, we cannot fully exclude the existence of an unknown, noncanonical pathway for acetate utilization that may be required to restore expression of these genes. We propose that the production of acetate by *C. jejuni* or the presence of acetate in the local intestinal environment may lead to expression of genes in *C. jejuni* required for commensal colonization of the avian host.

### Physiologically relevant SCFAs influence expression of acetogenesis-dependent genes.

Acetate is an SCFA metabolite produced as a by-product by commensal bacteria and is most abundantly found in the lower intestinal tracts of many hosts, including avian species (29, 30). Other SCFAs, such as butyrate and propionate, are also produced by commensals and commonly found in the same intestinal regions as acetate. In contrast, the metabolite lactate is often more abundant in the upper regions of the intestinal tract.

**FIG 7:**
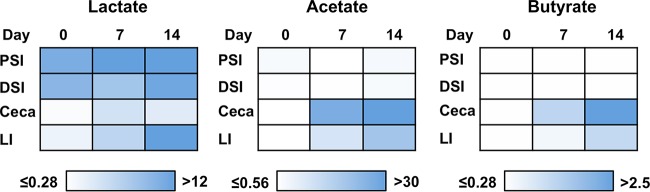
Quantification of lactate and SCFAs in the intestinal tracts of chicks over time. The levels of lactate and the acetate and butyrate SCFAs were measured in the contents of the proximal small intestine (PSI), distal small intestine (DSI), ceca, and large intestine (LI) in uninfected chicks on days 0, 7, and 14 after hatching by GC-MS. Six or seven chicks were processed at each time point. The geometric mean for the concentration of metabolite from each set of organs on each day was determined. Data are shown as a shade of blue respective to the heat map indicating a gradient of concentrations detected for each metabolite, with white to light blue representing the lowest concentrations and dark blue representing the highest concentrations. The numbers beside each heat map represent the lower and upper range of concentrations of each metabolite, indicated as millimoles per kilogram of organ content. The limit of detection for lactate and butyrate was 0.28 mmol/kg of organ content, and the limit of detection for acetate was 0.58 mmol/kg of organ content.

As discussed above, the *C. jejuni* Δ*pta ackA* mutant was dependent upon exogenous acetate for expression of the acetogenesis-dependent genes required for optimal commensal colonization. Additionally, this mutant displayed a colonization defect throughout the intestinal tracts of chicks when they were 7 days old, but this colonization defect was absent when they were 14 days old. Therefore, we hypothesized that the concentrations of acetate and perhaps other SCFAs produced by the microbiota might change with the age of chicks and may ultimately influence expression of the *C. jejuni* acetogenesis-dependent genes and the ability of *C. jejuni* to promote commensalism. In our chick model system, we were able to quantify the levels of lactate, acetate, and butyrate throughout the intestinal tracts of uninfected chicks from day 0 to 14 after hatching (Fig. 7 and Table S2). We found that lactate was present in chicks of all ages at a consistently high level in the proximal and distal small intestines (8 to 12 mmol/kg of content), but at much lower levels in the ceca and large intestines (Fig. 7). When the chicks were 14 days old, lactate accumulated in the large intestines. Acetate and butyrate levels in the proximal and distal small intestines in chicks of all ages or in the ceca and large intestines of chicks on the day they hatched (day 0) were either below or at the limit of detection (0.56 or 0.28 mmol/kg of content, respectively). In contrast, acetate and butyrate were much more abundant in the ceca and large intestines in 7-day-old or 14-day-old chicks. Our *in vivo* quantitation of lactate, acetate, and butyrate are in the lower ranges reported throughout the intestinal tract by others (29; reviewed in reference 30).

10.1128/mBio.00407-17.4 TABLE S2  *In vivo* levels of lactate, acetate, and butyrate in different regions of the intestinal tracts of uninfected chicks over time. Download TABLE S2, PDF file, 0.1 MB. Copyright © 2017 Luethy et al. 2017 Luethy et al. This content is distributed under the terms of the Creative Commons Attribution 4.0 International license.

**FIG 8:**
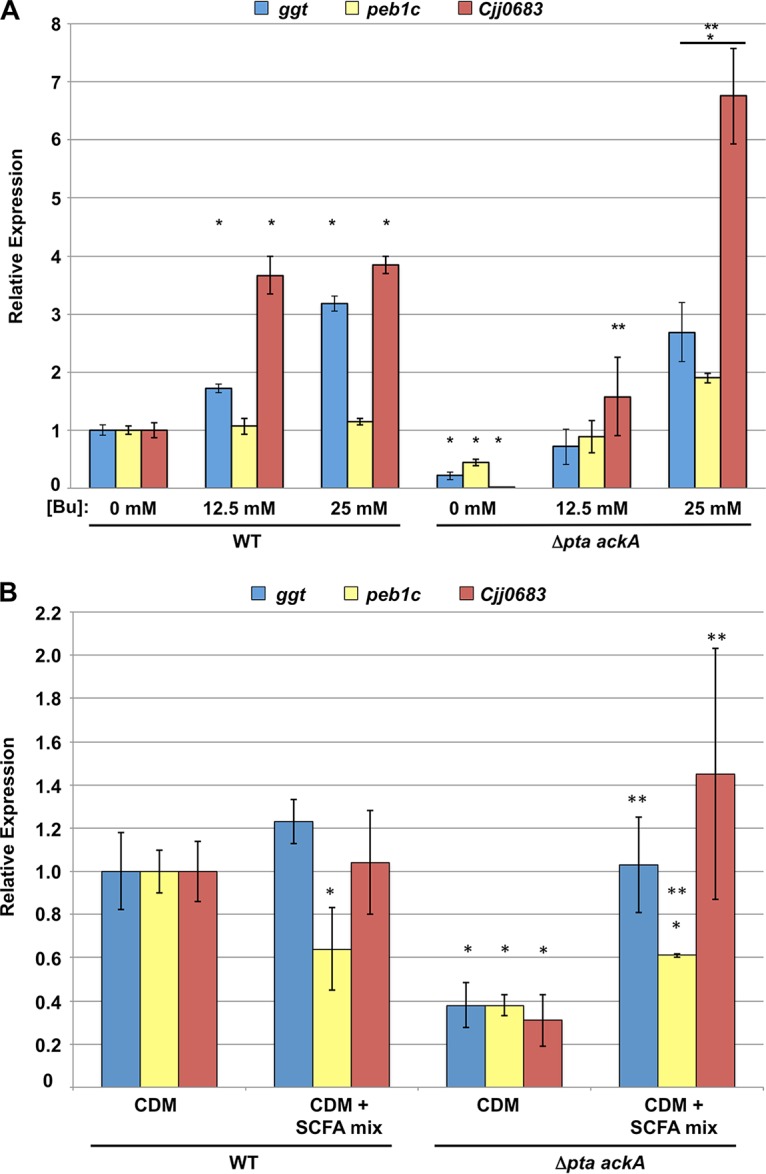
Effects of butyrate and an SCFA mixture on expression of acetogenesis-dependent genes in WT and *C. jejuni* Δ*pta ackA*. (A and B) Semiquantitative real-time PCR analysis of transcription of *ggt*, *peb1c*, and *Cjj0683* in WT *C. jejuni* and the isogenic Δ*pta ackA* mutant grown in CDM or CDM with different concentrations of butyrate (Bu) (A) or with a mixture of SCFAs at a final concentration of 30 mM acetate, 5 mM butyrate, and 7.5 mM propionate (B). All media were equilibrated to pH 7.0 prior to growth of bacteria to eliminate effects due to acidification by exogenous butyrate or SCFAs. The expression of *ggt*, *peb1c*, and *Cjj0683* in the WT *C. jejuni* 81-176 grown without any supplementation was measured by qRT-PCR and set at 1. The level of expression of each gene in the WT strain grown with supplementation or in the mutants grown with or without supplementation is shown relative to the WT strain grown without supplementation. Error bars indicate standard deviations. Statistically significant differences in gene expression between WT *C. jejuni* without supplementation and strains with or without supplementation (*, *P* < 0.05) or between each individual strain with or without supplementation (**, *P* < 0.05) as performed by Student’s *t* test are indicated.

We examined whether SCFAs other than acetate present in the avian lower intestinal tract could affect expression of the acetogenesis-dependent genes. Butyrate has been reported to be present in the ceca and lower intestines of chickens up to around 35 mM (29, 30). Addition of butyrate at 12.5 and 25 mM to WT *C. jejuni* caused up to a 3- to 4-fold increase in expression of two acetogenesis-dependent genes, *ggt* and *Cjj0683*, but *peb1c* expression remained unchanged (Fig. 8A). With *C. jejuni* Δ*pta ackA*, we observed a striking restoration of expression of all genes with 12.5 mM butyrate supplementation. The level of gene expression increased 2- to 100-fold in the Δ*pta ackA* mutant compared to the Δ*pta ackA* mutant without supplementation (Fig. 8A). With 25 mM butyrate supplementation to the Δ*pta ackA* mutant, we observed further stimulation that exceeded WT *C. jejuni* without butyrate by 2- to 7-fold. In comparison to *C. jejuni* Δ*pta ackA* stimulated with acetate, butyrate promoted a greater level of gene expression with a lower molar concentration, suggesting that *C. jejuni* is more sensitive to butyrate for stimulation of expression of these genes (compare Fig. 6B to 8A). We also noted that *C. jejuni* Δ*pta ackA* with 100 mM propionate supplementation restored expression of the acetogenesis-dependent genes to WT levels, although this level of propionate is 10- to 12-fold higher than has been reported in the chick ceca (Fig. S1) (29, 30).

10.1128/mBio.00407-17.2 FIG S1  Effect of propionate on expression of the acetogenesis-dependent genes in WT and *C. jejuni* Δ*pta ackA*. Semiquantitative real-time PCR analysis of transcription of *ggt*, *peb1c*, and *Cjj0683* in WT *C. jejuni* 81-176 Sm^r^ and isogenic Δ*pta ackA* mutants grown in CDM or CDM with different concentrations of propionate (Pr). All media were equilibrated to pH 7.0 prior to growth of bacteria to eliminate effects due to acidification by exogenous propionate. The expression of *ggt* (blue bars), *peb1c* (yellow bars), and *Cjj0683* (red bars) in the WT *C. jejuni* 81-176 Sm^r^ Δ*astA* grown without any supplementation was measured by qRT-PCR and set at 1. Expression of each gene in the WT strain growth with propionate supplementation or in the mutants grown with or without supplementation is shown relative to the WT strain grown without propionate supplementation. Error bars indicate standard deviations. Statistically significant differences in gene expression between WT *C. jejuni* without supplementation and strains with or without supplementation (*, *P* < 0.05) or between each individual strain with or without supplementation (**, *P* < 0.05) as performed by Student’s *t* test are indicated. Download FIG S1, PDF file, 0.3 MB. Copyright © 2017 Luethy et al. 2017 Luethy et al. This content is distributed under the terms of the Creative Commons Attribution 4.0 International license.

Unlike our *in vitro* system used above, *C. jejuni* is exposed to a mixture of SCFAs, including acetate, butyrate, and propionate, rather than only each individual SCFA, in the chick intestinal tract. Therefore, we analyzed whether a mixture of SCFAs in which each SCFA is present at a low concentration that is physiologically relevant and did not stimulate expression of these acetogenesis-dependent genes (Fig. 6B, 8A, and S1; data not shown) restored gene expression to the *C. jejuni* Δ*pta ackA* mutant. For this analysis, we supplemented CDM with an SCFA mixture containing 30 mM acetate, 5 mM butyrate, and 7.5 mM propionate. After growth of the WT strain with the SCFA mixture, expression of *ggt* and *Cjj0683* was unaffected, but a slight decrease was noted with *peb1c* (Fig. 8B). Compared to expression of these genes with the Δ*pta ackA* acetogenesis mutant grown in CDM, exposure to the SCFA mixture fully restored expression of *ggt* and *Cjj0683* to WT levels or above (Fig. 8B). *peb1c* expression also increased significantly when the Δ*pta ackA* mutant was grown in the SCFA mixture, although the levels did not increase to those of WT without the SCFA mixture. These results suggest that SCFAs can be sensed by *C. jejuni* and function together at low concentration to stimulate expression of the acetogenesis-dependent genes in the *C. jejuni* acetogenesis mutant.

### SCFAs and lactate influence expression of acetogenesis-dependent genes in opposing ways.

**FIG 9:**
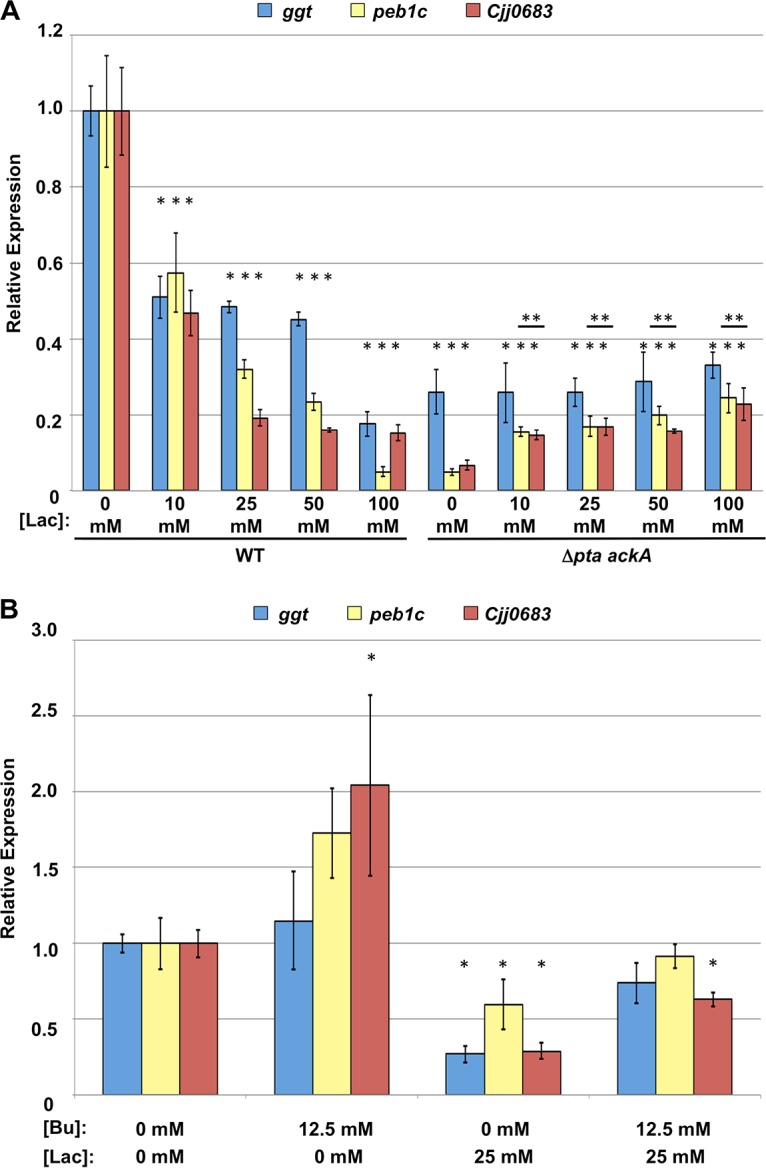
Effects of lactate and butyrate on expression of the acetogenesis-dependent genes in WT and *C. jejuni* Δ*pta ackA*. Semiquantitative real-time PCR analysis of transcription of *ggt*, *peb1c*, and *Cjj0683* in WT *C. jejuni* and isogenic Δ*pta ackA* mutants grown in CDM or CDM with different concentrations of lactate (Lac) (A) or a mixture of butyrate and lactate (B). In panel B, only the WT strain was analyzed with the indicated medium supplementations. All media were equilibrated to pH 7.0 prior to growth of bacteria to eliminate effects due to acidification by exogenous butyrate or lactate. The levels of expression of *ggt*, *peb1c*, and *Cjj0683* in the WT *C. jejuni* 81-176 grown without any supplementation were measured by qRT-PCR and set at 1. Expression of each gene in the WT strain grown with supplementation or mutants grown with or without supplementation is shown relative to the WT strain grown without supplementation. Error bars indicate standard deviations. Statistically significant differences in gene expression between WT *C. jejuni* without supplementation and strains with or without supplementation (*, *P* < 0.05) or between each individual strain with or without supplementation (**, *P* < 0.05) as performed by Student’s *t* test are indicated.

The organic acid lactate is present (~2 to 5 mmol/kg of content) in the avian lower intestinal tract but is found at much higher levels in the avian upper intestinal tract (~50 mmol/kg of content [30]). Because *C. jejuni* has a reduced ability to colonize the upper intestinal tract (compared to the lower intestinal tract) where lactate concentrations are high but SCFAs are low (Fig. 2 and 7), we tested whether lactate may impact and possibly reduce expression of the acetogenesis-dependent or SCFA-influenced genes in *C. jejuni*. After growth of WT *C. jejuni* in CDM with different concentrations of lactate, we observed a dose-dependent reduction of expression of these genes (Fig. 9A). We observed 2- to 5-fold reduction in gene expression with 25 mM lactate (Fig. 9A). For *C. jejuni* Δ*pta ackA*, we found very little effect of lactate supplementation on gene expression. We did observe up to a 3.5- to 6-fold increase in expression in *peb1c* and *Cjj0683*, but these increases were still 5-fold lower than WT *C. jejuni* without supplementation (Fig. 9A). These data indicate that lactate has an opposite effect on transcription of the SCFA-influenced genes in WT *C. jejuni*, with lactate possibly functioning *in vivo* to dampen expression of these genes in the upper regions of the intestinal tract where lactate is a predominant metabolite over SCFAs and *C. jejuni* colonizes less efficiently.

We next analyzed the net effect of a mixture of lactate and butyrate on gene expression in WT *C. jejuni*. For this assay, we supplemented CDM with physiological concentrations of 12.5 mM butyrate reported in the ceca and 25 mM lactate reported in the small intestines (29, 30). Consistent with our data described above, gene expression was stimulated with butyrate alone and repressed with lactate alone (Fig. 9B). However, in the presence of butyrate and lactate, the levels of expression of these genes were close to those of WT *C. jejuni* grown without either metabolite (Fig. 9B). These data suggest that lactate and SCFAs affect expression of these colonization genes in opposite ways. Furthermore, the data suggest that butyrate can counteract the repressive effects of lactate on expression of these genes and may function *in vivo* to stimulate expression of factors necessary for colonization, especially in the lower intestinal tract.

**FIG 10:**
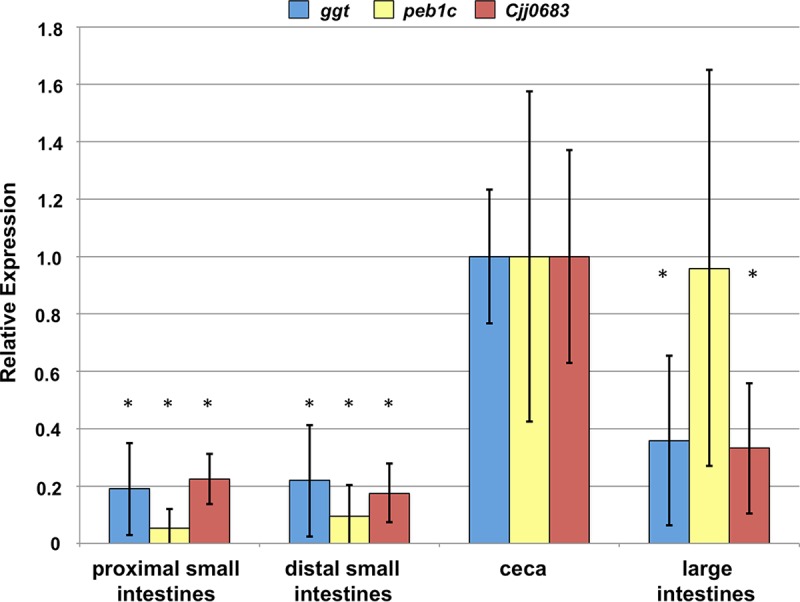
*In vivo* expression of the acetogenesis-dependent genes in *C. jejuni* colonizing different regions of the avian intestinal tract. Semiquantitative real-time PCR analysis of transcription of *ggt*, *peb1c*, and *Cjj0683* in WT *C. jejuni* isolated from different regions of the avian intestinal tract on day 7 postinfection. The levels of expression of *ggt*, *peb1c*, and *Cjj0683* in the WT *C. jejuni* 81-176 Sm^r^ isolated from the ceca as measured by qRT-PCR were set at 1. Expression of each gene in *C. jejuni* from different regions of the intestinal tract is shown relative to the *C. jejuni* isolated from the ceca. In total, gene expression was analyzed from six different chicks and combined. Error bars indicate standard deviations. Statistically significant differences in gene expression between WT *C. jejuni* isolated from the ceca and other regions of the intestinal tract (*, *P* < 0.05) as performed by Student’s *t* test are indicated.

We then investigated whether spatial differences in the expression of these genes by *C. jejuni* occurred during colonization of the chick gut. We reasoned that if lactate and SCFAs were impacting the *in vivo* expression of the SCFA-influenced genes in different intestinal regions, we may observe higher expression of these genes in the lower intestinal tract where SCFAs are prevalent than in the upper intestinal tract where lactate is abundant. Thus, we infected chicks on the day they hatched with WT *C. jejuni* and then monitored gene expression of WT *C. jejuni* colonizing different regions of the chick intestinal tract on day 7 postinfection. Expression of the SCFA-influenced genes in each region of the intestinal tract was determined relative to the expression of a control housekeeping gene, rather than relative to the absolute numbers of *C. jejuni* present at the site of colonization. The highest level of expression of the SCFA-influenced genes occurred in the ceca, which contains the highest loads of *C. jejuni* during colonization (Fig. 10 and 2A). In the large intestines, which contain the second highest levels of *C. jejuni*, we observed a 2.5-fold-lower decrease in expression of *ggt* and *Cjj0683*; the level of expression of *peb1c* on average was similar to *C. jejuni* in the ceca (Fig. 10). The levels of expression of these genes in the upper regions of the intestinal tract, including both regions of the small intestines, however, were 5- to 20-fold lower than those of *C. jejuni* in the ceca (Fig. 10). These data correlate with the highest level of expression of the SCFA-influenced genes occurring in regions of the avian intestinal tract with the highest levels of stimulating SCFAs and lowest levels of inhibitory lactate. Thus, we propose that *C. jejuni* monitors SCFAs in the environment to initiate production of catabolic pathways and colonization factors necessary for commensalism and persistence in favored niches in the natural avian host.

## DISCUSSION

In this work, we discovered that *C. jejuni* responds to common intestinal metabolites by altering transcription of genes required for commensal colonization of a natural avian host. These genes were first discovered to be dependent on the *C. jejuni* acetogenesis pathway for expression. Further analysis revealed that transcription of these acetogenesis-dependent genes was restored by supplementing an acetogenesis mutant with exogenous SCFAs, including acetate, butyrate, and propionate, with effects likely independent of SCFA metabolism. These SCFAs are often present in abundance in the lower regions of the intestinal tracts of avian and human hosts, which are the preferred niches for *C. jejuni* growth and colonization (29, 30, 62, 63). Expression of these SCFA-influenced genes was repressed by another intestinal metabolite, the organic acid lactate, which is usually abundant in the upper regions of the intestinal tract. This upper intestinal region supports less *C. jejuni* colonization. Furthermore, we provided *in vivo* correlative evidence that *C. jejuni* likely senses these metabolites throughout the intestinal tract of the natural avian host to modulate different levels of expression of the SCFA-influenced genes required for commensalism.

In the lower intestinal tract where SCFAs are abundant, *C. jejuni* expressed the highest levels of these SCFA-influenced genes and colonized efficiently. Many of these genes encode proteins for transport and utilization of amino acids that contribute to *C. jejuni* metabolism. *C. jejuni* has a great dependency on amino acids and keto acids rather than carbohydrates to supply carbon sources for metabolism. Thus, an SCFA-rich environment of the ceca and lower intestinal tract is likely recognized by *C. jejuni* and stimulates the bacterium to induce dissimilation and catabolic pathways needed for optimal growth and colonization in these preferred niches in the natural avian host. In contrast, the lowest levels of expression of the SCFA-influenced genes occurred in the small intestines, which contain higher concentrations of lactate. We propose that lactate, possibly with other metabolites in the upper regions of the avian intestinal tract, dampens gene expression for the SCFA-influenced colonization determinants. To the best of our knowledge, our work provides one of the first examples and strongest evidence for metabolites that are likely *in vivo* cues for *C. jejuni* that impact expression of colonization factors necessary for commensalism in the natural avian host. We propose that *C. jejuni* may discriminate between regions of the avian intestinal tract to find preferred niches for colonization by monitoring and responding to gradients of lactate and SCFAs in the avian host. We suspect that a similar spatial arrangement of lactate and SCFAs may be sensed by *C. jejuni* in the human host, which may lead to the production of virulence determinants to promote infection and diarrheal disease. Although SCFAs and lactate are some *in vivo* metabolites recognized by *C. jejuni*, the bacterium most likely monitors a combination of factors and conditions in the host gut to discern appropriate niches for colonization.

Considering that lactate and SCFAs are generated by the normal intestinal microbiota, our findings suggest that the microbiota indirectly spatially influences the ability of *C. jejuni*, another bacterial commensal in the avian intestine, to colonize different regions of the avian host. Our data also suggest that the microbiota can temporally influence the colonization dynamics of *C. jejuni* in the avian host. In this study, we orally gavaged chicks on the day they hatched with WT *C. jejuni* or the Δ*pta ackA* acetogenesis mutant and found that *C. jejuni* was dependent upon its own acetogenesis pathway for colonization throughout the intestinal tract at least through day 7 postinfection. However, by day 14 postinfection, the acetogenesis pathway was no longer required for colonization. Although we have not yet investigated the complexity of the microbiota in our chick model of commensalism, the data we acquired quantifying the levels of lactate, acetate, and butyrate throughout the chick intestines for up to 14 days indicate that the microbiota and microbiota-derived metabolites enrich in complexity over time. Furthermore, previous work analyzing the chick intestinal microbiota by others correlates with our findings that lactate-producing bacteria colonize after hatching of the chicks and dominate in the upper small intestines, with SCFA producers colonizing thereafter and dominating the ceca and lower intestinal tract (summarized in references 30 and 64). Thus, on the day the chicks hatch, the chicks at the time of infection in our assays possess an immature microbiota with lactate present but little to no SCFAs. The *in vivo* levels of SCFAs produced by the microbiota at this early time point of infection are likely not sufficient to be sensed by *C. jejuni* and activate expression of the SCFA-influenced genes. To compensate for the lack of SCFAs in these young chicks, *C. jejuni* apparently requires its acetogenesis pathway to generate acetate and stimulate expression of these genes necessary for optimal colonization. As the chicks age, the microbiota develops in complexity and contributes significant SCFA levels, especially in the ceca and large intestines. This surge of SCFAs appears to negate the need for the acetogenesis pathway of *C. jejuni* to supply acetate to maintain transcription of the SCFA-influenced genes sufficient for colonization in 14-day-old chicks. We propose that *C. jejuni* employs its acetogenesis system as a compensatory mechanism to ensure a supply of the SCFA acetate sufficient to induce expression of the SCFA-influenced genes. This compensatory mechanism to supply its own acetate appears to be especially important for *C. jejuni* if the bacterium is transmitted to a young chick with an immature microbiota community or in an intestinal region that lacks sufficient SCFA levels. New lines of investigation will focus on manipulating the avian intestinal microbiota relative to *C. jejuni* infection to strengthen our proposed correlations between SCFA and lactate generation, *in vivo* expression of the SCFA-influenced genes, and the commensal colonization capacity of *C. jejuni* in different regions of the avian intestinal tract.

We found that three different SCFAs, acetate, butyrate, and propionate, were each individually sufficient *in vitro* to restore expression of SCFA-influenced genes in the *C. jejuni* acetogenesis mutant. At least for acetate and butyrate, the levels of the SCFAs (50 to 100 mM and 12.5 mM, respectively) that restored expression of the genes to WT levels were at physiological concentrations in the chick ceca as reported by others (29, 30) and a modestly higher level than what we measured in our chick model. However, we were able to demonstrate that *C. jejuni* can sense a mixture of these SCFAs in which each SCFA is present at a level that did not restore gene expression when added individually. Additionally, the levels of the SCFAs in this mixture are mostly lower than those reported by us in our chick model or by others. Although we are not yet able to determine whether the effects of these SCFAs are additive or synergistic, it is clear that these SCFAs can together be sensed by *C. jejuni* and stimulate expression of the SCFA-influenced genes.

Currently, the mechanism by which *C. jejuni* may sense lactate and SCFAs to regulate transcription of SCFA-influenced genes is unknown. The *C. jejuni* Δ*pta ackA acs* mutant was reduced in expression of these genes and could be stimulated by acetate to express these genes at levels close to the WT levels. This mutant lacks all known pathways for catabolism of acetate, including conversion of acetate to Ac-CoA and Ac-P. Furthermore, butyrate (and propionate at a high concentration) also induced expression of these genes. Unlike other enteric bacteria, the *C. jejuni* chromosome lacks genes encoding any known proteins or enzymes for catabolism of butyrate or propionate. Considering this information and the data presented in this work, we strongly suggest that acetate, butyrate, and propionate serve as direct cues that influence one or more systems to induce expression of these SCFA-influenced genes. We attempted to determine whether a lactate dehydrogenase present in *C. jejuni* was required to promote the lactate-dependent repressive effects we observed, but we were unable to interpret the results because the data acquired from multiple mutants were inconsistent, perhaps due to pleiotropic effects upon mutation of these genes (data not shown). However, lactate dehydrogenase converts lactate to pyruvate, which is present in the CDM used in this study (24). Furthermore, serine, a favored amino acid in *C. jejuni* metabolism that is also present in CDM, is also converted to pyruvate by a serine dehydratase (18). In a preliminary assay where we added exogenous serine or pyruvate to 25 mM (the concentration of lactate that repressed expression of the SCFA-influenced genes) to the WT strain, transcription of these genes was unchanged compared to the WT strain without supplementation (data not shown). We propose that lactate conversion to pyruvate does not likely lead to repression of the SCFA-influenced gene and that lactate itself is the direct cue sensed by *C. jejuni* that leads to repression of gene expression. Considering that we observed effects of SCFAs and lactate on expression of SCFA-influenced genes and that the SCFAs and lactate could compete with each other for respective effects on transcription, the simplest explanation is that a system is present in *C. jejuni* to sense these metabolites as cues and activate or repress transcription of SCFA-influenced genes depending on the concentration and composition of lactate and SCFAs in the microenvironment. Although unlikely, it is possible that *C. jejuni* may contain novel and as yet uncharacterized pathways for catabolism of each of these SCFAs and lactate to convert each into a metabolite that is a cue to influence transcription of these genes.

In *Escherichia coli*, SCFAs are sensed by the BarA/SirA-UvrY two-component regulatory systems (39). SCFAs are detected by the BarA sensor kinase, which results in phosphorylation of the UvrY response regulator and transcription of the noncoding RNAs (ncRNAs) CsrB and CsrC. These ncRNAs sequester the mRNA-binding protein CsrA. Without sequestration, CsrA represses translation of mRNAs encoding proteins that function in various aspects of physiology such as carbon metabolism, motility, peptide uptake, and virulence. In *Salmonella*, acetate influences the BarA/SirA-UvrY system via Ac-P-dependent activation of SirA (40). However, propionate exerts its effects downstream of BarA/SirA-UvrY and the Csr system by influencing the activity and stability of the HilD transcriptional regulator to eventually repress genes required for invasion of eukaryotic cells (65). These studies also showed that while acetate activates expression of invasion genes, butyrate and propionate function to repress expression of invasion genes, demonstrating that different SCFAs have opposite effects on invasion gene expression (40, 65). Although *C. jejuni* produces CsrA, which affects expression and translation of some virulence and colonization genes, it lacks homologs of the BarA/UvrY system, CsrB, CsrC, and HilD (66–69). Thus, *C. jejuni* employs a different system than *E. coli* and *Salmonella* species to sense and respond to these metabolites in the chick intestinal tract.

We also noticed that not all SCFA-influenced genes were altered to the same degrees by lactate or SCFAs. Currently, without a transcriptional mechanism to explain how these metabolites influence expression of these genes, we are unable to comment on these differences in expression. We suspect that while there is an SCFA-dependent mechanism to influence expression of these genes, some of the genes may be affected by other systems and regulators to create complex mechanisms for their expression in different environments.

The repressive effects of lactate on the transcription of SCFA-influenced genes we observed shed new insights into how this metabolite may negatively impact *in vivo C. jejuni* growth and colonization. Lactate itself and lactate-producing bacteria are potential probiotics to reduce *C. jejuni* in poultry flocks in agriculture (70, 71). Administration of lactate to chicken carcasses after slaughter reduces the levels of *C. jejuni* on the surfaces of commercial poultry meats (72, 73). The potential antimicrobial properties of lactate remain to be elucidated. In one report, the acidification of the environment by lactate reduced the viability of *C. jejuni*, but evidence was presented for lactate having pH-independent effects on *C. jejuni* (70). In our assays where CDM was supplemented with lactate or any SCFAs, pH was not a factor, as all media were balanced to neutral pH. Considering our findings presented in this work, it is possible that a probiotic effect of lactate and lactate-producing bacteria is to lower transcription of genes required for *in vivo* growth and catabolism, thereby hindering *C. jejuni* colonization.

In this work, we identified dozens of SCFA-influenced genes where transcription is either stimulated by SCFAs or repressed by lactate. A fair number of genes with the greatest level of induction by SCFAs have previously been shown to be required for optimal levels of commensal colonization of avian species or infection of mice in different murine models, which indicate their potential to be required for virulence and diarrheal disease progression. We verified that *peb1c*, encoding a component of the Peb1 ABC transporter for uptake of glutamate and aspartate for catabolism of these amino acids, is required by *C. jejuni* for commensal colonization of the chick ceca (46). Many of the remaining genes have yet to be tested for commensal colonization of avian hosts or infection of mice. We suspect that this set of genes contains many more colonization and virulence factors that can influence interactions with different hosts. The combined reduction of expression of many of these factors likely contributes to the attenuation in colonization observed with the *C. jejuni* Δ*pta ackA* acetogenesis mutant throughout the intestinal tract in young chicks.

Our work provides strong evidence for metabolites, specifically lactate and SCFAs produced by the intestinal microbiota, to greatly impact the *in vivo* behavior of *C. jejuni* and its ability to colonize the natural avian host. We suspect that similar mechanisms may occur in the human host to impact the ability of *C. jejuni* to express virulence factors for pathogenesis of diarrheal disease in the human colon. Much future work will be required to unravel how lactate and SCFAs serve as cues for *C. jejuni* and how mechanisms of response to these cues lead to opposing effects on transcription of colonization and virulence determinants. Furthermore, our work may have implications for how lactate, and possibly other unidentified metabolites, may negatively impact *C. jejuni* growth *in vivo*, providing an opportunity to explore different antimicrobial strategies to reduce *C. jejuni* in the human host, agriculture, and the human food supply.

## MATERIALS AND METHODS

### Bacterial strains and plasmids.

All strains and plasmids used in this study are listed in Tables S3 and S4 in the supplemental material. *C. jejuni* strains were routinely grown from freezer stocks under microaerobic conditions (10% CO_2_, 5% O_2_ and 85% N_2_) on Mueller-Hinton (MH) agar containing 10 μg/ml trimethoprim at 37°C for 48 h. For analysis of *C. jejuni* strains grown in the presence of specific SCFAs, *Campylobacter* defined medium (CDM) was used and supplemented with one or more short-chain fatty acids (SCFAs) at a specific concentration. The medium contains all 20 amino acids (ranging from 5 to 19 mM) and the keto acids α-ketoglutarate and pyruvate (5 to 9 mM) as primary carbon sources (34). Strains were then restreaked onto appropriate media and grown for an additional 16 h. The following antibiotics were added to the media, when appropriate, at the concentrations indicated: 100 μg/ml kanamycin; 20 μg/ml chloramphenicol; 30 μg/ml cefoperazone; or 0.1, 0.5, 1, 2, or 5 mg/ml streptomycin. *E. coli* DH5α strains were grown on LB agar or in LB broth containing 100 μg/ml ampicillin, 100 μg/ml kanamycin, or 12.5 μg/ml tetracycline, when necessary. *C. jejuni* strains were stored at −80°C in a mixture of 85% MH broth and 15% glycerol. *E. coli* strains were stored at −80°C in a mixture of 80% LB broth and 20% glycerol.

10.1128/mBio.00407-17.5 TABLE S3  Bacterial strains used in this study. Download TABLE S3, PDF file, 0.1 MB. Copyright © 2017 Luethy et al. 2017 Luethy et al. This content is distributed under the terms of the Creative Commons Attribution 4.0 International license.

10.1128/mBio.00407-17.6 TABLE S4  Plasmids used in this study. Download TABLE S4, PDF file, 0.1 MB. Copyright © 2017 Luethy et al. 2017 Luethy et al. This content is distributed under the terms of the Creative Commons Attribution 4.0 International license.

### Construction of *C. jejuni* mutants.

*C. jejuni* mutants were constructed through electroporation using previously published methods (74). Briefly, the use of pJMB553 (pUC19::*pta ackA* [28]) facilitated the creation of strains with mutations in the *pta ackA* locus. In order to create a *pta* deletion, a *cat-rpsL* resistance cassette was removed from pDRH265 (74) through SmaI digestion and ligated into the SpeI site of *pta* in pJMB533, resulting in the creation of pJMB566. Electroporation of pJMB566 into *C. jejuni* 81-176 Sm^r^ Δ*astA* (DRH461) and recovery of transformants on MH agar plates containing chloramphenicol resulted in the creation of strain JMB611 (81-176 Sm^r^ Δ*astA pta*::*cat-rpsL*) (33, 74). To cleanly remove the *pta* gene from the *C. jejuni* genome, an in-frame deletion of *pta* was created on pJMB553 through PCR-mediated mutagenesis (75). DNA sequencing verified the mutation, resulting in the plasmid pJMB627 (pUC19::Δ*pta*). The plasmid was electroporated into strain JMB611, and transformants were recovered on MH agar containing 0.5 to 5.0 mg/ml streptomycin. The deletion of *pta* was verified using colony PCR, resulting in the creation of strain JMB638 (81-176 Sm^r^ Δ*astA pta*).

Mutants lacking the *ackA* gene were created as previously described (28). Briefly, the plasmid pJMB653 (containing *ackA*::*cat-rpsL*) was electroporated into strain DRH461. The transformants were recovered on MH agar plates containing chloramphenicol to result in strain PML1239 (81-176 Sm^r^ Δ*astA ackA*::*cat-rpsL*), which was confirmed by colony PCR. A mutation resulting in the gutting of both the *pta* and *ackA* genes was performed as previously described, resulting in plasmid pJMB955 (28). pJMB955 was electroporated into strain DRH461, and transformants were recovered on MH agar plates containing chloramphenicol, resulting in the creation of strain JMB957 (81-176 Sm^r^ Δ*astA pta ackA*::*cat-rpsL*), which was confirmed by colony PCR.

The *ggt*, *peb1C*, *Cjj0682-0683*, *acs*, *pycA*, and *gltA* loci were amplified via PCR from the *C. jejuni* 81-176 chromosome using primers located approximately 750 bp upstream and downstream of the locus and containing 5′ BamHI sites. The PCR products were cloned into the BamHI site of pUC19 to create pPML456 (pUC19::*ggt*), pPML706 (pUC19::*Cjj0682-0683*), pPML725 (pUC19::*peb1C*), pPML874 (pUC19::*gltA*), pPML963 (pUC19::*acs*), and pABT255 (pUC19::*pycA*). An EcoRV site was created in *Cjj0683* (on pPML706) through PCR-mediated mutagenesis to create pPML708 (75, 76). A *cat-rpsL* resistance cassette was removed from pDRH265 by SmaI digestion and ligated into the EcoRV sites of *ggt* and *Cjj0683* and into the HpaI site of *peb1C*, resulting in the creation of pPML1013, pPML1014, and pPML1017, respectively (74). A *kan-rpsL* resistance cassette was removed from pDRH437 by SmaI digestion and ligated into the AfeI site of *acs* to create pPML1001, the BsaBI site of *gltA* to create pPML925, and into the T4 DNA polymerase filled-in NcoI site of *pycA* to create pPML941 (33). Plasmids pPML1013, pPML1014, and pPML1017 were subsequently electroporated into strain DRH212, and transformants were recovered on MH agar plates containing chloramphenicol. Strains PML1049 (81-176 Sm^r^ *peb1c*::*cat-rpsL*), PML1059 (81-176 Sm^r^ *ggt*::*cat-rpsL*), and PML1065 (81-176 Sm^r^ *Cjj0683*::*cat-rpsL*) were recovered and confirmed by colony PCR. Plasmids pPML1001, pPML925, and pPML941 were electroporated into strains DRH461 (81-176 Sm^r^ Δ*astA*) and JMB957 (81-176 Sm^r^ Δ*astA pta ackA*::*cat-rpsL*), and transformants were recovered on MH agar containing kanamycin, resulting in strains PML953 (81-176 Sm^r^ Δ*astA gltA*::*kan-rpsL*), PML956 (81-176 Sm^r^ Δ*astA pycA*::*kan-rpsL*), PML960 (81-176 Sm^r^ Δ*astA pta ackA*::*cat-rpsL pycA*::*kan-rpsL*), PML1006 (81-176 Sm^r^ Δ*astA acs*::*kan-rpsL*), and PML1009 (81-176 Sm^r^ Δ*astA pta ackA*::*cat-rpsL acs*::*kan-rpsL*). Mutants were confirmed by colony PCR.

To remove the *Cjj0683* gene from the *C. jejuni* genome, an in-frame deletion of *Cjj0683* was created on pPML706 through PCR-mediated mutagenesis (75). DNA sequencing verified the mutation, resulting in the plasmid pPML1144 (pUC19::Δ*Cjj0683*). The plasmid was electroporated into strain PML1065, and transformants were recovered on MH agar containing 0.5-5.0 mg/ml streptomycin. The deletion of *Cjj0683* was verified using colony PCR, resulting in the creation of strain PML1160 (81-176 Sm^r^ Δ*Cjj0683*).

### Construction of plasmids for complementation of *C. jejuni* in *trans*.

To overexpress the *pta* and *ackA* genes in *trans* from a plasmid in *C. jejuni*, a fragment containing the 203 bp upstream of the start codon of *flaA* followed by the start codon and an NcoI site was amplified by PCR using primers with a 5′ XbaI site and a 3′ BamHI site. Following restriction digestion, this fragment was inserted into the corresponding sites in pRY108 to create pDAR1423 (pRY108::P*flaA*-NcoI). A 2.7-kb fragment, including codon 2 of *pta* through codon 396 of *ackA* was amplified by PCR using primers with 5′ and 3′ NcoI sites. The fragment was digested with NcoI and inserted into the NcoI site in pDAR1423 to create pPML1071 (pDAR1423::*pta ackA*).

pPML1071 and pDAR1423 were transformed into chemically competent *E. coli* DH5α/pRK212.1 which contains the conjugation transfer element (77) and conjugated into the appropriate *C. jejuni* strains (77, 78). Transconjugants were recovered on MH agar containing kanamycin, streptomycin, and trimethoprim, and the presence of the plasmid was verified by colony PCR.

### Chick colonization assays.

All use of animals in experimentation has been approved by the IACUC at the University of Texas Southwestern Medical Center. The ability of WT or isogenic mutant 81-176 Sm^r^ *C. jejuni* strains to colonize the ceca of chicks after oral inoculation was determined as previously described (5). Briefly, fertilized chicken eggs (SPAFAS) were incubated for 21 days at 37.8°C with appropriate humidity and rotation in a Sportsman II model 1502 incubator (Georgia Quail Farms Manufacturing Company). Within 24 h of hatching, chicks were orally inoculated with 100 μl of MH broth containing approximately 10^2^ CFU of either the WT or mutant strain. Strains were prepared for infection by suspending *C. jejuni* strains from MH agar plates after 16 h of growth at 37°C and microaerobic conditions in MH broth and diluting the strains to achieve the appropriate inoculum for oral gavage of the chicks. Dilutions of the inoculum were plated on MH agar to assess the number of bacteria in each inoculum. At 7 or 14 days postinfection, chicks were sacrificed. The proximal small intestine, distal small intestine, cecal, or large intestine contents were removed and suspended in phosphate-buffered saline (PBS), and serial dilutions were plated on MH agar containing trimethoprim and cefoperazone. Following 72 h of growth at 37°C under microaerobic conditions, the bacteria were counted to determine the number of CFU per gram of cecal contents. The small intestine was divided in half by length to create what we term the proximal and distal small intestine.

### Determination of *in vivo* lactate and SCFA concentrations.

Fertilized eggs were incubated as described above until the chicks hatched. Uninfected chicks were divided into groups of 6 or 7 chicks and given heat, water, and food *ad libitum*. On days 0, 7, and 14 after hatching, the chicks were sacrificed, and the contents of the proximal small intestine, distal small intestine, ceca, and large intestine from each chick were removed. The contents were immediately resuspended in PBS to a final concentration of 200 mg/ml. Samples were vortexed to disrupt particulate matter and then centrifuged at 6,000 rpm for 10 min to pellet any remaining debris. For each sample, 85 μl was combined with 15 μl of a solution containing deuterated acetate, lactate, and butyrate so that each deuterated metabolite was at a final concentration of 50 μM. Samples were dried without heat in a Speed-Vac and then stored at −80°C until use.

Dried extracts were solubilized by sonication in 0.1 ml anhydrous pyridine and then incubated for 20 min at 80°C. An equal amount of *N-tert*-butyldimethylsilyl-*N*-methyltrifluoroacetamide with 1% *tert*-butyldimethylchlorosilate (Sigma-Aldrich) was added, and the samples were incubated for 1 h at 80°C. Samples were centrifuged at 20,000 × *g* for 1 min to remove particles. One hundred microliters of the supernatant was transferred to an autosampler vial and analyzed by gas chromatography-mass spectrometry (Shimadzu TQ8040 mass spectrometer). The sample was injected with a 1:50 split ratio at an injection temperature of 250°C on an Rtx 5 Sil mass spectrometer (MS) (30-m-length, 0.25-mm-diameter, 0.25-μm-film-thickness) fused silica capillary column. Helium was used as the carrier gas with a constant velocity of 50 cm/s. The gas chromatograph (GC) oven temperature started at 50°C for 2 min, rising to 100°C at 20°C/min and holding for 3 min, then raised to 330°C at 40°C/min with a final hold for 3 min. The interface was heated to 300°C. The ion source was used in electron ionization (EI) mode (70 V, 150 μA, 200°C). The event time for selected ion monitoring (SIM) and multiple reaction monitoring (MRM) events was 50 ms. The mass spectrometry method, monitored *m/z* (quantifier in italics), and experimentally determined retention indices (RI) (*N*-alkane scale) are as follows: acetate (SIM, *117*, 75, 159; RI, 949), butyrate-d_7_ (SIM, *152*, 76, 153; RI, 1081), butyrate (SIM, *145*, 75, 146; RI, 1086), lactate-d_3_ (MRM, *264* > *236*, collision energy (CE) 6 V; 264 > 189, CE 8 V; RI, 1470), lactate (MRM, *261* > *233*, CE 6 V; 261 > 189, CE 8 V; RI, 1478). Efficient recovery of target metabolites was determined using deuterated compounds as internal standards. Quantification was based on external standards comprised of a series of dilutions of pure compounds, derivatized as described above at the same time as the samples.

### Determination of acetate concentrations *in vitro*.

WT *C. jejuni* 81-176 Sm^r^ Δ*astA* (DRH461) or isogenic *pta* and *ackA* mutants were grown from freezer stocks in MH agar containing trimethoprim at 37°C under microaerobic conditions for 48 h. Strains were then restreaked on MH agar and grown for an additional 16 h. *C. jejuni* growth was suspended from the plates in CDM broth and diluted into 25 ml of CDM broth containing trimethoprim to an optical density at 600 nm (OD_600_) of approximately 0.1 (34). Cultures were grown at 37°C under microaerobic conditions without shaking to mid-log phase. Samples of cultures for analysis were taken every 4 h. Cultures were centrifuged for 5 min at 13,000 rpm to pellet cells, and the supernatant was removed. The supernatant was then centrifuged again for 5 min at 13,000 rpm to remove any remaining *C. jejuni* cells. Triplicate samples from the supernatants of *C. jejuni* DRH461 (WT) and JMB957 (*pta ackA*::*cat-rpsL*) were used as directed in a microplate assay for detection of acetic acid (Megazyme), with uninoculated CDM broth serving as a control.

### Growth curve analysis in MH and CDM broth.

*C. jejuni* strains PML1140, PML1125, and PML1102 were grown from freezer stocks on MH agar containing kanamycin at 37°C under microaerobic conditions for 48 h. Strains were then restreaked on MH agar containing kanamycin and grown for an additional 16 h. *C. jejuni* growth was suspended from the plates in MH or CDM broth and diluted into 25 ml of MH or CDM broth containing trimethoprim and kanamycin to an OD_600_ of approximately 0.1 (34). Strain PML1140 served as the WT control, PML1125 served as the *pta ackA* mutant, and PML1102 was a *pta ackA* complemented strain. Cultures were grown at 37°C under microaerobic conditions without shaking, and growth was measured via absorbance at 600 nm from 0 h up to 80 h. Each culture was grown in triplicate.

### Collection of RNA for microarray analysis.

WT *C. jejuni* 81-176 Sm^r^ (DRH212) and the isogenic Δ*astA pta ackA*::*cat-rpsL* (JMB957) mutant were grown from freezer stocks on MH agar containing trimethoprim at 37°C under microaerobic conditions for 48 h. Strains were then restreaked on MH agar and grown for an additional 16 h. *C. jejuni* growth was suspended from the plates in MH broth and diluted into 25 ml of MH broth to an OD_600_ of approximately 0.05. Strains were then grown statically at 37°C under microaerobic conditions for 8 h to achieve mid-log-phase growth. Following growth, strains were suspended in 10× stop solution (95% ethanol plus 5% phenol), incubated on ice for 20 min, pelleted by centrifugation, and stored at −80°C (79). Pellets were suspended in 1 ml of RiboZol (Amresco), and RNA was removed by chloroform extraction. Total RNA (60 μg) was treated with DNase I (Invitrogen) and then purified through an RNeasy Mini Column (Qiagen). Real-time PCR (RT-PCR) was performed to confirm the absence of DNA from the RNA samples.

### Transcriptome analysis with DNA microarrays.

A DNA microarray-based transcriptome analysis was performed as previously described (80, 81). Briefly, comparisons of gene expression were performed indirectly by comparing the transcriptome profile of WT *C. jejuni* 81-176 Sm^r^ Δ*astA* (DRH461) or 81-176 Sm^r^ Δ*astA pta ackA*::*cat-rpsL* (JMB957). Total RNA samples (20 μg) for each strain were labeled with Cy3-dUTP during cDNA production by reverse transcriptase and mixed with Cy5-dUTP-labeled reference genomic DNA from WT *C. jejuni* 81-176 Sm^r^ before being hybridized separately to custom cDNA microarrays (Gene Expression Omnibus [GEO] platform GPL6315 on Corning UltraGAPS slides). Microarrays were scanned using an Axon GenePix 4000B microarray laser scanner (Axon Instruments, Union City, CA). Microarray experiments were performed with two technical replicates per array, with two replicate features for each coding sequence per array. GenePix 4.0 software was used to process the spot and background intensities, and data normalization was performed to compensate for differences in the amount of template or unequal Cy3 or Cy5 dye incorporation. GeneSpring 7.3 software (Silicon Genetics, Palo Alto, CA) was used to analyze the normalized data, and the parametric statistical *t* test was used to determine the significance of the centered data (*P* values of <0.05 indicate statistical significance), with adjustment of the individual *P* values by use of the Benjamini-Hochberg false-discovery rate multiple-test correction in the GeneSpring analysis package.

### Semiquantitative real-time RT-PCR analysis.

WT *C. jejuni* and isogenic mutant strains were grown from freezer stocks on MH agar containing trimethoprim at 37°C under microaerobic conditions for 48 h. Strains were then restreaked on MH agar and grown for an additional 16 h. *C. jejuni* growth was suspended from the plates in MH broth and diluted into 25 ml of MH broth to an OD_600_ of approximately 0.1. Strains were then grown statically at 37°C under microaerobic conditions for 8 h to achieve mid-log-phase growth. Total RNA was extracted with RiboZol (Amresco), and RNA was treated with DNase I (Invitrogen). RNA was diluted to a concentration of 50 ng/μl before analysis. Semiquantitative real-time PCR (qRT-PCR) was performed using a 7500 real-time PCR system (Applied Biosystems) with *secD* mRNA detection as an endogenous control. mRNA transcript levels in strains DRH212 and DRH461 served as WT controls to determine relative gene expression in isogenic mutants.

To examine the effects of different short-chain fatty acids on gene expression in *C. jejuni*, WT and isogeneic mutant strains were grown from freezer stocks on MH agar as detailed above. Following the additional 16 h of growth, the *C. jejuni* strains were suspended from plates in CDM, which is a medium containing nutrients at specific concentrations to support growth, and diluted into 25 ml of CDM broth to an OD_600_ of approximately 0.1 (34). Strains were grown in CDM alone, CDM containing 0, 10, 25, 50, or 100 mM potassium acetate, sodium propionate, or sodium l-lactate (Sigma), CDM containing 0, 12.5, or 25 mM sodium butyrate (Acros Organics), or CDM containing 12.5 mM sodium butyrate and 25 mM sodium l-lactate. For analysis of how a mixture of SCFAs impacts gene expression, strains were grown in CDM alone or CDM containing 30 mM potassium acetate, 5 mM sodium butyrate, and 7.5 mM sodium propionate. All media were adjusted to pH 7.0 prior to inoculation of bacteria. Bacterial growth occurred statically at 37°C under microaerobic conditions for 8 h to achieve mid-log-phase growth. Total RNA was extracted with RiboZol (Amresco), and RNA was treated with DNase I (Invitrogen). RNA was diluted to a concentration of 50 ng/μl before analysis. qRT-PCR was performed using a 7500 real-time PCR system (Applied Biosystems) with *secD* mRNA detection as an endogenous control. mRNA transcript levels in the DRH461 strain grown in CDM alone served as the WT control to determine relative gene expression in the wild type and isogenic mutant at the different concentrations of SCFAs.

To examine the effect of overexpression in *trans* of *pta* and *ackA* on gene expression in *C. jejuni*, strains PML1102, PML1125, and PML1140 were grown in MH and CDM broth containing kanamycin and trimethoprim. Total RNA was extracted and purified as detailed above. mRNA transcript levels in strain PML1140 were used as the WT control, and *secD* mRNA detection was used as the endogenous control to determine relative gene expression.

To examine the levels of expression of colonization and virulence genes throughout the chick intestinal tract, 1-day-old chicks were orally inoculated with 100 μl of MH broth containing approximately 10^2^ CFU of WT *C. jejuni* as described above. At 7 days postinfection, the chicks were sacrificed, the small intestine, cecal, or large intestine contents were removed, and total RNA was extracted with RiboZol (Amresco). RNA was then purified as described above. mRNA transcript levels in the ceca served as the organ load control, and *secD* mRNA detection was used as the endogenous control to determine relative gene expression.

### Availability of data.

Data from microarray analyses were submitted to the GEO database with series accession number GSE86327 and are available for review.

10.1128/mBio.00407-17.1 TEXT S1  Supplemental references. Download TEXT S1, PDF file, 0.1 MB. Copyright © 2017 Luethy et al. 2017 Luethy et al. This content is distributed under the terms of the Creative Commons Attribution 4.0 International license.
