# Supplementary material for: Microbiota-Derived Short-Chain Fatty Acids Modulate Expression of Campylobacter jejuni Determinants Required for Commensalism and Virulence
Source: mBio. 2017 May 9;8(3):e00407-17. doi: 10.1128/mBio.00407-17 (PMC5424204; doi:10.1128/mBio.00407-17)
Supplement: TABLE S1 [file mbo002173300st1.pdf]

Table S1. Complete list of genes differentially expressed in *C. jejuni* 81-176 Sm^{R} Δ*pta ackA* compared to WT *C. jejuni* 81-176 Sm^{Ra}

Locus Tag	Gene Name	Putative Function	Ratio WT/ mutant	Reference
81176_0711	*pta*	phosphotransacetylase	82.74	(1)
81176_0711	*ackA*	acetate kinase	10.02	(1)
81176_0038	*rrc*	Rbo/RbR-like protein; rubrerythrin-like protein	9.50	(2)
81176_0067	*ggt*	γ-glutamyltransferase	8.11	(3-6)
81176_0204		hypothetical protein	7.99	
81176_0122	*aspA*	aspartate ammonia-lyase	7.51	(7, 8)
81176_0056	*ansA*	L-asparginase	7.28	(4)
81176_0292		putative cytochrome c-type heme-binding protein	6.58	
81176_0697	*dcuB*	C4-dicarboxylate transporter	6.19	(7)
81176_0683		putative di-/tripeptide transporter	5.90	
81176_0928	*peb1a*	amino acid transporter; periplasmic binding protein	5.48	(9-12)
81176_0123	*dcuA*	C4-dicarboxylate transporter	5.22	(7)
81176_0929	*peb1c*	amino acid transporter; ATP-binding protein	5.11	(9-12)
81176_0474		hypothetical protein	5.05	
81176_0291		trimethylamine N-oxide (TMAO) reductase	5.02	(13)
81176_1731		DNA-binding protein HU homolog	4.99	(14)
81176_0440		hypothetical protein	4.99	
81176_1358		hypothetical integral membrane protein	4.90	
81176_1198	*cfa*	cyclopropane fatty acyl phospholipid synthase	4.65	
81176_0382		cytochrome c551 peroxidase	4.49	(15)
81176_0743	*flaC*	flagellin-like protein	4.38	(16, 17)
81176_1170		cytochrome-related hypothetical protein	4.19	
81176_0443		hypothetical protein	4.08	
81176_0107		hypothetical protein	4.07	
81176_0393	*rpsU*	30S ribosomal protein S21	4.06	
81176_0880	*dsbA*	thiol:disulfide interchange protein	3.92	(18)
81176_0464	*mfrB*	methylmenaquinol:fumarate protein B	3.89	(19)
81176_0465	*mfrC*	methylmenaquinol:fumarate protein C	3.73	(19)
81176_0037		putative ComEA-related protein	3.73	(20)
81176_0473		methyl-accepting chemotaxis protein Tlp6	3.52	(21)
81176_0641	*cft*	ferritin	3.47	(22)
81176_0881	*dsbB*	disulfide bond formation protein B	3.42	(18)
81176_0974		hypothetical protein	3.39	
81176_0682		pseudogene; putative	3.38	
81176_1519		hypothetical protein	3.37	
81176_0917		hypothetical protein	3.32	

81176_1242	*htrA*	periplasmic serine protease	3.21	(23, 24)
81176_0463	*mfrA*	methylmenaquinol:fumarate protein A	3.20	(19)
81176_0448		hypothetical protein	3.17	
81176_1675	*gltA*	citrate synthase	3.12	
81176_0624	*peb4*	cell binding factor 2 precursor; major antigenic peptide	3.03	(25, 26)
81176_1382		probable periplasmic protein	3.03	
81176_0415	*pyk*	pyruvate kinase	2.99	(27)
81176_1016		hypothetical protein	2.89	
81176_1457	*flgM*	anti-σ factor for σ^{28}	2.88	(28-30)
81176_0758		hypothetical protein	2.88	
81176_1525	*tupA*	tungstate ABC transporter; substrate-binding protein	2.81	(31)
81176_1443	*ciaI*	probable ATP/GTP binding protein; invasion and colonization protein	2.79	(32-34)
81176_1338	*flaA*	major flagellin	2.77	(35)
81176_0432	*frdC*	fumarate reductase and succinate dehydrogenase; cytochrome b subunit	2.76	(19, 36, 37)
81176_1616	*sdaC*	L-serine transporter	2.75	(11, 38)
81176_0927	*peb1b*	amino acid transporter; permease protein	2.74	
81176_1179		hypothetical protein	2.72	
81176_0591		hypothetical protein	2.67	
81176_0416	*mqo*	predicted malate:quinone oxidoreductase	2.67	
81176_1615	*sdaA*	L-serine dehydratase	2.66	(38)
81176_1257	*ciaC*	invasion protein	2.60	(39, 40)
81176_0577		hypothetical integral membrane protein	2.58	
81176_0950		hypothetical protein	2.52	
81176_0434	*frdB*	fumarate reductase and succinate dehydrogenase; iron-sulfur protein	2.43	(19, 36, 37)
81176_0113	*lctP*	L-lactate permease	2.43	(41)
81176_0764		putative outer membrane protein	2.43	
81176_1139	*wlaK*	putative aminotransferase	2.42	
81176_0446		putative ABC transporter; ATP-binding protein	2.42	
81176_0383		phosphoglucomutase/phosphor-mannomutatse family protein	2.39	
81176_0722	*glnA*	glutamine synthase	2.37	
81176_1444	*dut*	putative deoxyuridinetriphosphatase	2.35	
81176_0048		putative fumarylacetoacetate hydrolase family protein	2.35	
81176_0907		HIT family hydrolase	2.33	
81176_0783	*aspB*	aspartate aminotransferase	2.32	(7)
81176_0869	*hemL*	glutamate-1-semialdehyde 2,1-aminomutase	2.32	
81176_0392		ferredoxin domain-containing	2.31	

		integral membrane protein		
81176_0414	*fedB*	secreted flagellar co-expressed determinant required for chick colonization	2.25	(33, 34)
81176_1485		hypothetical membrane protein	2.23	
81176_0553	*flgB*	flagellar rod protein	2.21	
81176_1213	*luxS*	autoinducer-2 production protein	2.20	(42)
81176_1471	*cadF*	fibronectin-binding protein	2.19	(43-45)
81176_0852	*acnB2*	aconitate hydrolase 2	2.18	
81176_1093	*fliW*	flagellar chaperone protein	2.15	(46-48)
81176_1526		LamB/YscF family protein	2.15	
81176_0397		2-hydroxyacid dehydrogenase family protein	2.14	
81176_0620		hypothetical protein	2.14	
81176_0572	*flaG*	possible flagellar protein	2.13	
81176_1494	*putP*	sodium/proline symporter	2.13	(11)
81176_0556	*icd*	isocitrate dehydrogenase	2.11	
81176_1207	*dctA*	C4-dicarboxylate transport protein	2.10	
81176_1339	*flaB*	minor flagellin	2.06	(35)
81176_0720	*flgF*	flagellar rod protein	2.06	
81176_1402	*gapA*	glyceraldehyde-3-phosphate dehydrogenase	2.06	
81176_0953		hypothetical protein	2.01	

81176_0315	*peb3*	glycoprotein; putative adhesion or transport protein	0.22	(49-51)
81176_1088		hypothetical protein	0.25	
81176_0643	*pstC*	phosphate ABC transporter; permease protein	0.27	(52)
81176_1087		hypothetical protein	0.32	
81176_1387	*katA*	catalase	0.33	(53)
81176_1435		putative sugar transferase	0.35	
81176_0515		hypothetical protein	0.36	
81176_0001	*rplC*	50S ribosomal protein L3	0.39	
81176_0644	*pstA*	phosphate ABC transporter; permease protein	0.39	(52)
81176_0752		hypothetical protein	0.39	
81176_1301	*trkA*	putative TRK system potassium protein	0.40	
81176_1699	*rplV*	50S ribosomal protein L22	0.40	
81176_1569	*dppA*	peptide ABC transporter; periplasmic substrate-binding protein	0.41	
81176_0642	*pstS*	phosphate ABC transporter; periplasmic phosphate binding protein	0.42	(52)
81176_0585		putative integral membrane protein	0.42	
81176_0076		hypothetical protein	0.42	

81176_1388		ankyrin repeat family protein	0.42	
81176_1620	*exbD2*	TolR/ExbD family biopolymer transport protein	0.43	(54)
81176_0549		hypothetical membrane protein; possible sodium-dependent transporter	0.44	
81176_0616	*thyA*	hemolysin A	0.45	
81176_0878	*pabB*	para-aminobenzoate synthase; glutamine amidotransferase component I	0.45	
81176_0005	*ksgA*	dimethyladenosine transferase	0.46	
81176_1697	*rplP*	50S ribosomal protein L16	0.46	
81176_1246		hypothetical protein	0.46	
81176_1512		hypothetical protein	0.46	
81176_1702	*rplW*	50S ribosomal protein L23	0.47	
81176_0977		hypothetical protein; DnaJ homolog	0.47	
81176_1694	*rplN*	50S ribosomal protein L14	0.47	
81176_0119	*cydB*	cytochrome bd oxidase subunit II	0.47	
81176_1029		hypothetical protein	0.47	
81176_0715		hypothetical membrane protein	0.48	
81176_0118	*cydA*	cytochrome bd oxidase subunit I	0.49	
81176_0004		putative beta-lactamase family protein	0.49	
81176_1703	*rplD*	50S ribosomal protein L4	0.50	
81176_1655		possible periplasmic thiredoxin	0.50	
81176_1059		probable membrane transport protein	0.50	
81176_1619	*exbB2*	MotA/TolQ/ExbB family biopolymer transport protein	0.50	(54)

[a] Expression of genes was increased or decreased by two-fold in the *C. jejuni* Δ*pta ackA*
