# Supplementary material for: Microbiota-Derived Short-Chain Fatty Acids Modulate Expression of Campylobacter jejuni Determinants Required for Commensalism and Virulence
Source: mBio. 2017 May 9;8(3):e00407-17. doi: 10.1128/mBio.00407-17 (PMC5424204; doi:10.1128/mBio.00407-17)
Supplement: TABLE S2 [file mbo002173300st2.pdf]

Table S2. *In vivo* Levels of Lactate, Acetate, and Butyrate in Different Regions of the Intestinal Tracts of Uninfected Chicks Over Time[a]

Organ	Day Post-Hatch	Lactate (mmol/kg)		Acetate (mmol/kg)		Butyrate (mmol/kg)	
		Range	Geo Mean ± SD	Range	Geo Mean ± SD	Range	Geo Mean ± SD
Proximal Small Intestines	0	8.0–12.3	10.8±1.5	1.7-3.7	2.6±0.6	<0.28[b]	<0.28±0
	7	11.4-13.7	12.8±1.0	<0.56-0.9[b]	<0.6±0.1	<0.28[b]	<0.28±0
	14	12.1-13.7	13.2±0.8	1.6-4.9	2.7±1.7	<0.28[b]	<0.28±0
Distal Small Intestines	0	6.9-12.4	9.8±2.3	<0.56-2.3[b]	<1.2±0.7	<0.28[b]	<0.28±0
	7	6.4-9.8	7.9±1.4	<0.56-1.3[b]	<0.9±0.3	<0.28[b]	<0.28±0
	14	11.6±0.9	1.6±5.3	1.6-5.3	2.5±1.3	<0.28[b]	<0.28±0
Ceca	0	<0.28-5.2[b]	<0.9±2.1	<0.56[b]	<0.56±0	<0.28[b]	<0.28±0
	7	2.3-10.5	4.2±2.9	20.5-40.3	26.8±7.0	0.4-5.6	1.3±1.9
	14	1.0-16.2	2.9±5.3 2	7.4-40.3	30.4±6.1	1.0-7.0	2.5±2.0
Large Intestines	0	<0.28-6.9[b]	<2.0±2.6	<0.56-0.9[b]	<0.6±0.1	<0.28b	<0.28±0
	7	2.0-10.8	5.9±3.4	2.7-23.2	9.5±7.8	<0.28-1.5[b]	<0.5±0.5
	14	9.6-15.3	12.3±2.3	9.4-42.2	19.5±10.3	0.4-3.3	1.2±1.0

[a] Concentrations of lactate, acetate, and butyrate were measured in the contents of each organ of a chick by GC-MS as described in the Experimental Procedures. Each group contained 6 or 7 chicks. Data are reported as a range or geometric mean +/- standard deviation in mmol/kg units.
[b] One or more chicks in this group contained the metabolite at a level below the limit of detection.
