# Supplementary material for: Microbiota-Derived Short-Chain Fatty Acids Modulate Expression of Campylobacter jejuni Determinants Required for Commensalism and Virulence
Source: mBio. 2017 May 9;8(3):e00407-17. doi: 10.1128/mBio.00407-17 (PMC5424204; doi:10.1128/mBio.00407-17)
Supplement: FIG S1 [file mbo002173300sf1.pdf]

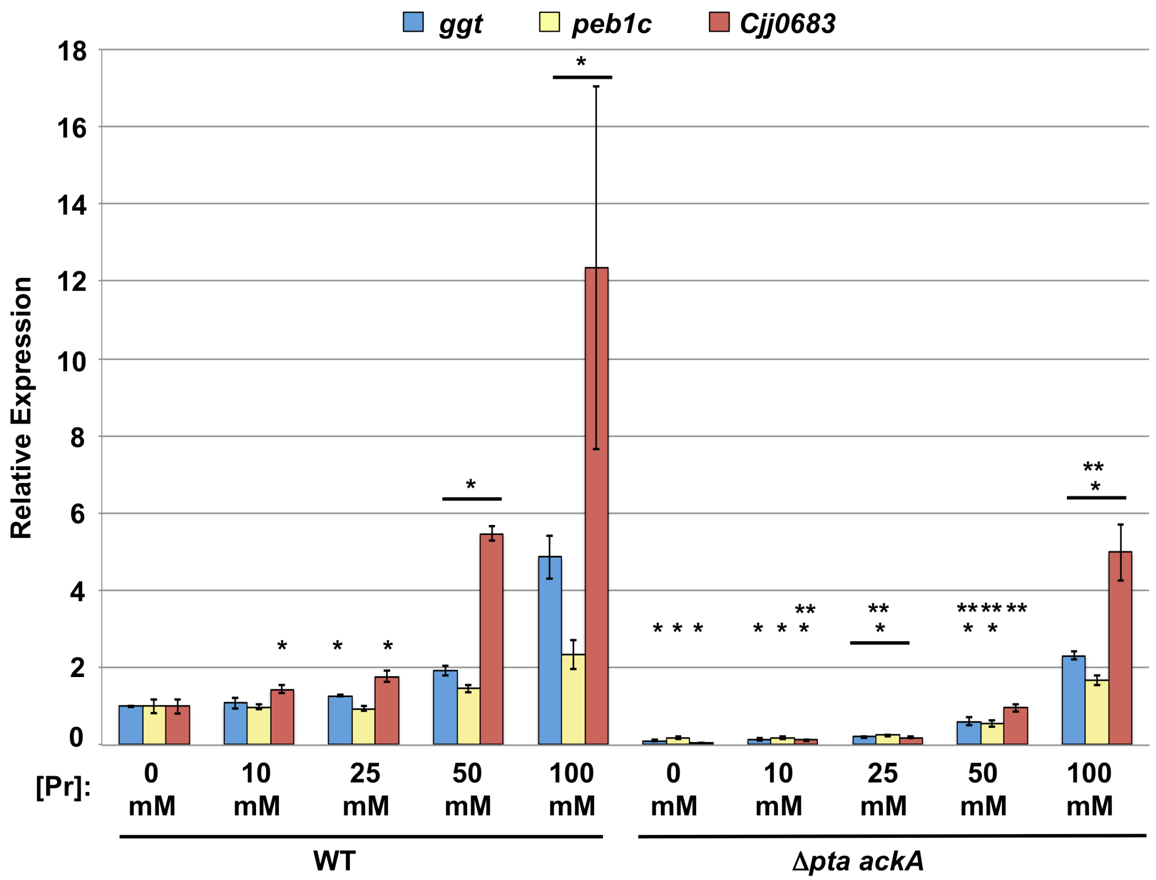

Figure S1. Effect of propionate on expression of the acetogenesis-dependent regulon in WT and *C. jejuni* Δ*pta ackA*. Semi-quantitative real-time PCR analysis of transcription of *ggt*, *peb1c*, and *Cjj0683* in WT *C. jejuni* and isogenic Δ*pta ackA* mutants grown in CDM or CDM with different concentrations of propionate (Pr). All media was equilibrated to pH 7.0 prior to growth of bacteria to eliminate effects due to acidification by exogenous propionate. The expression of *ggt* (blue bars), *peb1c* (yellow bars), and *Cjj0683* (red bars) in the WT *C. jejuni* 81-176 grown without any supplementation was measured by qRT-PCR was set to 1. Expression of each gene in the mutants grown with or without supplementation is shown relative to the WT strain. Error bars indicate standard deviations. Statistically significant differences in gene expression between WT *C. jejuni* without supplementation and strains with or without supplementation (*, $P < 0.05$) or between each individual strain without with or without supplementation (**, $P < 0.05$) as performed by the Student's *t*-test are indicated.
