# Supplementary material for: Microbiota-Derived Short-Chain Fatty Acids Modulate Expression of Campylobacter jejuni Determinants Required for Commensalism and Virulence
Source: mBio. 2017 May 9;8(3):e00407-17. doi: 10.1128/mBio.00407-17 (PMC5424204; doi:10.1128/mBio.00407-17)
Supplement: TABLE S3 [file mbo002173300st3.pdf]

Table S3. Bacterial strains used in this study

Strain	Genotype	Source/Reference
DH5α	*E. coli supE44* Δ*lac*U169 (ϕ80*lacZ*ΔM15) *hsdR*17 *recA*1 *endA*1 *gyrA*96 *thi*-1 *relA*1	Invitrogen
DH5α/RK212.1	DH5α with conjugation transfer element	(55)
81-176	Wild-type *C. jejuni* clinical isolate	(56)
DRH212	81-176 $rpsL^{Sm}$	(57)
DRH461	DRH212 Δ*astA*	(30)
JMB611	DRH461 *pta*::*cat-rpsL*	This study
JMB638	DRH461 Δ*pta*	This study
JMB957	DRH461 *pta ackA::cat-rpsL*	This study
PML1006	DRH461 *acs*::*kan-rpsL*	This study
PML1009	JMB957 *acs*::*kan-rpsL*	This study
PML1049	DRH212 *peb1C*::*cat-rpsL*	This study
PML1059	DRH212 *ggt*::*cat-rpsL*	This study
PML1065	DRH212 *Cjj0683*::*cat-rpsL*	This study
PML1102	JMB957 / pPML1071	This study
PML1125	JMB957 / pDAR1423	This study
PML1140	DRH461 / pDAR1423	This study
PML1160	DRH461 Δ*Cjj0683*	This study
PML1239	DRH461 *ackA*::*cat-rpsL*	This study
