# Supplementary material for: Microbiota-Derived Short-Chain Fatty Acids Modulate Expression of Campylobacter jejuni Determinants Required for Commensalism and Virulence
Source: mBio. 2017 May 9;8(3):e00407-17. doi: 10.1128/mBio.00407-17 (PMC5424204; doi:10.1128/mBio.00407-17)
Supplement: TABLE S4 [file mbo002173300st4.pdf]

Table S4. Plasmids used in this study

Plasmid	Genotype/Description	Source/Reference
pBR322	Amp^r Tet^r	New England Biolabs
pUC19	Amp^r	New England Biolabs
pRY108	Km^R; *E.coli*-*C. jejuni* shuttle vector	(58)
pDRH265	pUC19::*cat-rpsL*	(57)
pDRH436	pUC19::*kan-rpsL*	(30)
pJMB553	pUC19::*pta ackA*	(59)
pJMB566	pJMB553 with *cat-rpsL* inserted into the SpeI site of *pta*	This study
pJMB627	pJMB553 with an in-frame deletion of codons 2-510 of *pta*	This study
pJMB653	pUC19 containing *ackA*::*cat-rpsL*	(59)
pJMB955	pUC19 containing *pta ackA*::*cat-rpsL*	(59)
pDAR1423	pRY108 with the promoter and start codon of *flaA* from 81-176 cloned into the XbaI and BamHI sites with an additional NcoI site between the *flaA* start codon and the BamHI site	This study
pPML456	pUC19 with the *ggt* locus from 81-176 cloned into the BamHI site	This study
pPML706	pUC19 with the *Cjj0682-0683* locus from 81-176 cloned into the BamHI site	This study
pPML708	pPML706 with a mutation to generate an EcoRV site in *Cjj0683*	This study
pPML725	pUC19 with the *peb1C* locus from 81-176 cloned into the BamHI site	This study
pPML963	pUC19 with the *acs* locus from 81-176 cloned into the BamHI site	This study
pPML1001	pPML963 with *kan-rpsL* cloned into the AfeI site of *acs*	This study
pPML1013	pPML456 with *cat-rpsL* cloned into the EcoRV site of *ggt*	This study
pPML1014	pPML708 with *cat-rpsL* cloned into the EcoRV site of *Cjj0683*	This study
pPML1017	pPML725 with *cat-rpsL* cloned into the HpaI site of *peb1C*	This study
pPML1071	pDAR1423 with a 2.7 kb segment including the coding sequences of *pta* and *ackA* cloned into the NcoI site	This study
PML1144	pPML706 with an in-frame deletion of codons 2-123 of *Cjj0683*	This study
