# Supplementary material for: Microbiota-Derived Short-Chain Fatty Acids Modulate Expression of Campylobacter jejuni Determinants Required for Commensalism and Virulence
Source: mBio. 2017 May 9;8(3):e00407-17. doi: 10.1128/mBio.00407-17 (PMC5424204; doi:10.1128/mBio.00407-17)
Supplement: TEXT S1 [file mbo002173300s1.pdf]

References

1. **Wright JA, Grant AJ, Hurd D, Harrison M, Guccione EJ, Kelly DJ, Maskell DJ.** 2009. Metabolite and transcriptome analysis of *Campylobacter jejuni in vitro* growth reveals a stationary-phase physiological switch. Microbiology **155:**80-94.
2. **Yamasaki M, Igimi S, Katayama Y, Yamamoto S, Amano F.** 2004. Identification of an oxidative stress-sensitive protein from *Campylobacter jejuni*, homologous to rubredoxin oxidoreductase/rubrerythrin. FEMS Microbiol Lett **235:**57-63.
3. **Barnes IH, Bagnall MC, Browning DD, Thompson SA, Manning G, Newell DG.** 2007. Gamma-glutamyl transpeptidase has a role in the persistent colonization of the avian gut by *Campylobacter jejuni*. Microb Pathog **43:**198-207.
4. **Hofreuter D, Novik V, Galan JE.** 2008. Metabolic diversity in *Campylobacter jejuni* enhances specific tissue colonization. Cell Host Microbe **4:**425-433.
5. **Vorwerk H, Mohr J, Huber C, Wensel O, Schmidt-Hohagen K, Gripp E, Josenhans C, Schomburg D, Eisenreich W, Hofreuter D.** 2014. Utilization of host-derived cysteine-containing peptides overcomes the restricted sulphur metabolism of *Campylobacter jejuni*. Mol Microbiol **93:**1224-1245.
6. **Hofreuter D, Tsai J, Watson RO, Novik V, Altman B, Benitez M, Clark C, Perbost C, Jarvie T, Du L, Galan JE.** 2006. Unique features of a highly pathogenic *Campylobacter jejuni* strain. Infect Immun **74:**4694-4707.
7. **Guccione E, Leon-Kempis Mdel R, Pearson BM, Hitchin E, Mulholland F, van Diemen PM, Stevens MP, Kelly DJ.** 2008. Amino acid-dependent growth of *Campylobacter jejuni*: key roles for aspartase (AspA) under microaerobic and oxygen-limited conditions and identification of AspB (Cj0762), essential for growth on glutamate. Mol Microbiol **69:**77-93.
8. **Novik V, Hofreuter D, Galan JE.** 2010. Identification of *Campylobacter jejuni* genes involved in its interaction with epithelial cells. Infect Immun **78:**3540-3553.
9. **Pei Z, Burucoa C, Grignon B, Baqar S, Huang XZ, Kopecko DJ, Bourgeois AL, Fauchere JL, Blaser MJ.** 1998. Mutation in the *peb1A* locus of *Campylobacter jejuni* reduces interactions with epithelial cells and intestinal colonization of mice. Infect Immun **66:**938-943.
10. **Leon-Kempis Mdel R, Guccione E, Mulholland F, Williamson MP, Kelly DJ.** 2006. The *Campylobacter jejuni* PEB1a adhesin is an aspartate/glutamate-binding protein of an ABC transporter essential for microaerobic growth on dicarboxylic amino acids. Mol Microbiol **60:**1262-1275.
11. **Hofreuter D, Mohr J, Wensel O, Rademacher S, Schreiber K, Schomburg D, Gao B, Galan JE.** 2012. Contribution of amino acid catabolism to the tissue specific persistence of *Campylobacter jejuni* in a murine colonization model. PLoS One **7:**e50699.
12. **Flanagan RC, Neal-McKinney JM, Dhillon AS, Miller WG, Konkel ME.** 2009. Examination of *Campylobacter jejuni* putative adhesins leads to the identification of a new protein, designated FlpA, required for chicken colonization. Infect Immun **77:**2399-2407.
13. **Sellars MJ, Hall SJ, Kelly DJ.** 2002. Growth of *Campylobacter jejuni* supported by respiration of fumarate, nitrate, nitrite, trimethylamine-*N*-oxide, or dimethyl sulfoxide requires oxygen. J Bacteriol **184:**4187-4196.
14. **Konkel ME, Marconi RT, Mead DJ, Cieplak W, Jr.** 1994. Cloning and expression of the hup encoding a histone-like protein of *Campylobacter jejuni*. Gene **146:**83-86.

15. **Bingham-Ramos LK, Hendrixson DR.** 2008. Characterization of two putative cytochrome c peroxidases of *Campylobacter jejuni* involved in promoting commensal colonization of poultry. Infect Immun **76:**1105-1114.
16. **Song YC, Jin S, Louie H, Ng D, Lau R, Zhang Y, Weerasekera R, Al Rashid S, Ward LA, Der SD, Chan VL.** 2004. FlaC, a protein of *Campylobacter jejuni* TGH9011 (ATCC43431) secreted through the flagellar apparatus, binds epithelial cells and influences cell invasion. Mol Microbiol **53:**541-553.
17. **Faber E, Gripp E, Maurischat S, Kaspers B, Tedin K, Menz S, Zuraw A, Kershaw O, Yang I, Rautenschlein S, Josenhans C.** 2016. Novel Immunomodulatory Flagellin-Like Protein FlaC in *Campylobacter jejuni* and Other Campylobacterales. mSphere **1**:e00028.
18. **Grabowska AD, Wywial E, Dunin-Horkawicz S, Lasica AM, Wosten MM, Nagy-Staron A, Godlewska R, Bocian-Ostrzycka K, Pienkowska K, Laniewski P, Bujnicki JM, van Putten JP, Jagusztyn-Krynicka EK.** 2014. Functional and bioinformatics analysis of two *Campylobacter jejuni* homologs of the thiol-disulfide oxidoreductase, DsbA. PLoS One **9:**e106247.
19. **Guccione E, Hitchcock A, Hall SJ, Mulholland F, Shearer N, van Vliet AH, Kelly DJ.** 2010. Reduction of fumarate, mesaconate and crotonate by Mfr, a novel oxygen-regulated periplasmic reductase in *Campylobacter jejuni*. Environ Microbiol **12:**576-591.
20. **Jeon B, Zhang Q.** 2007. Cj0011c, a periplasmic single- and double-stranded DNA-binding protein, contributes to natural transformation in *Campylobacter jejuni*. J Bacteriol **189:**7399-7407.
21. **Chandrashekhar K, Gangaiah D, Pina-Mimbela R, Kassem, II, Jeon BH, Rajashekara G.** 2015. Transducer like proteins of *Campylobacter jejuni* 81-176: role in chemotaxis and colonization of the chicken gastrointestinal tract. Front Cell Infect Microbiol **5:**46.
22. **Wai SN, Nakayama K, Umene K, Moriya T, Amako K.** 1996. Construction of a ferritin-deficient mutant of *Campylobacter jejuni*: contribution of ferritin to iron storage and protection against oxidative stress. Mol Microbiol **20:**1127-1134.
23. **Brondsted L, Andersen MT, Parker M, Jorgensen K, Ingmer H.** 2005. The HtrA protease of *Campylobacter jejuni* is required for heat and oxygen tolerance and for optimal interaction with human epithelial cells. Appl Environ Microbiol **71:**3205-3212.
24. **Boehm M, Hoy B, Rohde M, Tegtmeyer N, Baek KT, Oyarzabal OA, Brondsted L, Wessler S, Backert S.** 2012. Rapid paracellular transmigration of *Campylobacter jejuni* across polarized epithelial cells without affecting TER: role of proteolytic-active HtrA cleaving E-cadherin but not fibronectin. Gut Pathog **4:**3.
25. **Zhang M, Meng F, Cao F, Qiao B, Liu G, Liu H, Zhou Y, Dong H, Gu Y, Xiao D, Zhang Y, Zhang J.** 2012. Cloning, expression, and antigenicity of 14 proteins from *Campylobacter jejuni*. Foodborne Pathog Dis **9:**706-712.
26. **Kale A, Phansopa C, Suwannachart C, Craven CJ, Rafferty JB, Kelly DJ.** 2011. The virulence factor PEB4 (Cj0596) and the periplasmic protein Cj1289 are two structurally related SurA-like chaperones in the human pathogen *Campylobacter jejuni*. J Biol Chem **286:**21254-21265.
27. **Velayudhan J, Kelly DJ.** 2002. Analysis of gluconeogenic and anaplerotic enzymes in *Campylobacter jejuni*: an essential role for phosphoenolpyruvate carboxykinase. Microbiology **148:**685-694.

28. **Wosten MM, van Dijk L, Veenendaal AK, de Zoete MR, Bleumink-Pluijm NM, van Putten JP.** 2010. Temperature-dependent FlgM/FliA complex formation regulates *Campylobacter jejuni* flagella length. Mol Microbiol **75:**1577-1591.
29. **Wosten MMSM, Wagenaar JA, van Putten JPM.** 2004. The FlgS/FlgR two-component signal transduction system regulates the *fla* regulon in *Campylobacter jejuni*. J Biol Chem **279:**16214-16222.
30. **Hendrixson DR, DiRita VJ.** 2003. Transcription of σ^{54}-dependent but not σ^{28}-dependent flagellar genes in *Campylobacter jejuni* is associated with formation of the flagellar secretory apparatus. Mol Microbiol **50:**687-702.
31. **Smart JP, Cliff MJ, Kelly DJ.** 2009. A role for tungsten in the biology of *Campylobacter jejuni*: tungstate stimulates formate dehydrogenase activity and is transported via an ultra-high affinity ABC system distinct from the molybdate transporter. Mol Microbiol **74:**742-757.
32. **Buelow DR, Christensen JE, Neal-McKinney JM, Konkel ME.** 2011. *Campylobacter jejuni* survival within human epithelial cells is enhanced by the secreted protein CiaI. Mol Microbiol **80:**1296-1312.
33. **Barrero-Tobon AM, Hendrixson DR.** 2012. Identification and analysis of flagellar coexpressed determinants (Feds) of *Campylobacter jejuni* involved in colonization. Mol Microbiol **84:**352-369.
34. **Barrero-Tobon AM, Hendrixson DR.** 2014. Flagellar biosynthesis exerts temporal regulation of secretion of specific *Campylobacter jejuni* colonization and virulence determinants. Mol Microbiol **93:**957-974.
35. **Guerry P, Alm RA, Power ME, Logan SM, Trust TJ.** 1991. Role of two flagellin genes in *Campylobacter* motility. J Bacteriol **173:**4757-4764.
36. **Smith MA, Mendz GL, Jorgensen MA, Hazell SL.** 1999. Fumarate metabolism and the microaerophily of *Campylobacter* species. Int J Biochem Cell Biol **31:**961-975.
37. **Weingarten RA, Taveirne ME, Olson JW.** 2009. The dual-functioning fumarate reductase is the sole succinate:quinone reductase in *Campylobacter jejuni* and is required for full host colonization. J Bacteriol **191:**5293-5300.
38. **Velayudhan J, Jones MA, Barrow PA, Kelly DJ.** 2004. L-serine catabolism via an oxygen-labile L-serine dehydratase is essential for colonization of the avian gut by *Campylobacter jejuni*. Infect Immun **72:**260-268.
39. **Christensen JE, Pacheco SA, Konkel ME.** 2009. Identification of a *Campylobacter jejuni*-secreted protein required for maximal invasion of host cells. Mol Microbiol **73:**650-662.
40. **Neal-McKinney JM, Konkel ME.** 2012. The *Campylobacter jejuni* CiaC virulence protein is secreted from the flagellum and delivered to the cytosol of host cells. Front Cell Infect Microbiol **2:**31.
41. **Thomas MT, Shepherd M, Poole RK, van Vliet AH, Kelly DJ, Pearson BM.** 2011. Two respiratory enzyme systems in *Campylobacter jejuni* NCTC 11168 contribute to growth on L-lactate. Environ Microbiol **13:**48-61.
42. **Adler L, Alter T, Sharbati S, Golz G.** 2014. Phenotypes of *Campylobacter jejuni luxS* mutants are depending on strain background, kind of mutation and experimental conditions. PLoS One **9:**e104399.

43. **Konkel ME, Garvis SG, Tipton SL, Anderson DE, Jr, Cieplak W, Jr.** 1997. Identification and molecular cloning of a gene encoding a fibronectin-binding protein (CadF) from *Campylobacter jejuni*. Mol Microbiol **24:**953-963.
44. **Monteville MR, Yoon JE, Konkel ME.** 2003. Maximal adherence and invasion of INT 407 cells by *Campylobacter jejuni* requires the CadF outer-membrane protein and microfilament reorganization. Microbiology **149:**153-165.
45. **Ziprin RL, Young CR, Stanker LH, Hume ME, Konkel ME.** 1999. The absence of cecal colonization of chicks by a mutant of *Campylobacter jejuni* not expressing bacterial fibronectin-binding protein. Avian Dis **43:**596-589.
46. **Dugar G, Svensson SL, Bischler T, Waldchen S, Reinhardt R, Sauer M, Sharma CM.** 2016. The CsrA-FliW network controls polar localization of the dual-function flagellin mRNA in *Campylobacter jejuni*. Nat Commun **7:**11667.
47. **Fields JA, Li J, Gulbronson CJ, Hendrixson DR, Thompson SA.** 2016. *Campylobacter jejuni* CsrA regulates metabolic and virulence associated proteins and is necessary for mouse colonization. PLoS One **11:**e0156932.
48. **Radomska KA, Ordonez SR, Wosten MM, Wagenaar JA, van Putten JP.** 2016. Feedback control of *Campylobacter jejuni* flagellin levels through reciprocal binding of FliW to flagellin and the global regulator CsrA. Mol Microbiol (in press).
49. **Linton D, Allan E, Karlyshev AV, Cronshaw AD, Wren BW.** 2002. Identification of *N*-acetylgalactosamine-containing glycoproteins PEB3 and CgpA in *Campylobacter jejuni*. Mol Microbiol **43:**497-508.
50. **Min T, Vedadi M, Watson DC, Wasney GA, Munger C, Cygler M, Matte A, Young NM.** 2009. Specificity of *Campylobacter jejuni* adhesin PEB3 for phosphates and structural differences among its ligand complexes. Biochemistry **48:**3057-3067.
51. **Young NM, Brisson JR, Kelly J, Watson DC, Tessier L, Lanthier PH, Jarrell HC, Cadotte N, St Michael F, Aberg E, Szymanski CM.** 2002. Structure of the *N*-linked glycan present on multiple glycoproteins in the Gram-negative bacterium, *Campylobacter jejuni*. J Biol Chem **277:**42530-42539.
52. **Wosten MM, Parker CT, van Mourik A, Guilhabert MR, van Dijk L, van Putten JP.** 2006. The *Campylobacter jejuni* PhosS/PhosR operon represents a non-classical phosphate-sensitive two-component system. Mol Microbiol **62:**278-291.
53. **Grant KA, Park SF.** 1995. Molecular characterization of *katA* from *Campylobacter jejuni* and generation of a catalase-deficient mutant of *Campylobacter coli* by interspecific allelic exchange. Microbiology **141:**1369-1376.
54. **Zeng X, Xu F, Lin J.** 2013. Specific TonB-ExbB-ExbD energy transduction systems required for ferric enterobactin acquisition in *Campylobacter*. FEMS Microbiol Lett **347:**83-91.
55. **Figurski DH, Helinski DR.** 1979. Replication of an origin-containing derivative of plasmid RK2 dependent on a plasmid function provided in trans. Proc Natl Acad Sci U S A **76:**1648-1652.
56. **Korlath JA, Osterholm MT, Judy LA, Forfang JC, Robinson RA.** 1985. A point-source outbreak of campylobacteriosis associated with consumption of raw milk. J Infect Dis **152:**592-596.
57. **Hendrixson DR, Akerley BJ, DiRita VJ.** 2001. Transposon mutagenesis of *Campylobacter jejuni* identifies a bipartite energy taxis system required for motility. Mol Microbiol **40:**214-224.

58. **Yao R, Alm RA, Trust TJ, Guerry P.** 1993. Construction of new *Campylobacter* cloning vectors and a new mutational *cat* cassette. Gene **130:**127-130.
59. **Boll JM, Hendrixson DR.** 2011. A specificity determinant for phosphorylation in a response regulator prevents in vivo cross-talk and modification by acetyl phosphate. Proc Natl Acad Sci U S A **108:**20160-20165.
